# Speed of lung inflation at birth influences the initiation of lung injury in preterm lambs

**DOI:** 10.1172/jci.insight.181228

**Published:** 2024-08-06

**Authors:** David G. Tingay, Monique Fatmous, Kelly Kenna, Jack Chapman, Ellen Douglas, Arun Sett, Qi Hui Poh, Sophia I. Dahm, Tuyen Kim Quach, Magdy Sourial, Haoyun Fang, David W. Greening, Prue M. Pereira-Fantini

**Affiliations:** 1 Neonatal Research, Murdoch Children’s Research Institute, Parkville, Australia.; 2 Department of Paediatrics, University of Melbourne, Melbourne, Australia.; 3 Newborn Services, Joan Kirner Women’s and Children’s, Sunshine Hospital, Western Health, St Albans, Australia.; 4 Department of Obstetrics and Gynaecology, University of Melbourne, Melbourne, Australia.; 5 Translational Research Unit, Murdoch Children’s Research Institute, Parkville, Australia.; 6 Molecular Proteomics, Baker Heart and Diabetes Institute, Melbourne, Australia.; 7 Baker Department of Cardiometabolic Health, The University of Melbourne, Melbourne, Australia.; 8 Baker Department of Cardiovascular Research, Translation and Implementation, La Trobe University, Melbourne, Australia.

**Keywords:** Inflammation, Pulmonology, Hypoxia, Pulmonary surfactants, Respiration

## Abstract

Gas flow is fundamental for driving tidal ventilation and, thus, the speed of lung motion, but current bias flow settings to support the preterm lung after birth do not have an evidence base. We aimed to determine the role of gas bias flow rates to generate positive pressure ventilation in initiating early lung injury pathways in the preterm lamb. Using slower speeds to inflate the lung during tidal ventilation (gas flow rates 4–6 L/min) did not affect lung mechanics, mechanical power, or gas exchange compared with those currently used in clinical practice (8–10 L/min). Speed of pressure and volume change during inflation were faster with higher flow rates. Lower flow rates resulted in less bronchoalveolar fluid protein, better lung morphology, and fewer detached epithelial cells. Overall, relative to unventilated fetal controls, there was greater protein change using 8-10 L/min, which was associated with enrichment of acute inflammatory and innate responses. Slowing the speed of lung motion by supporting the preterm lung from birth with lower flow rates than in current clinical use resulted in less lung injury without compromising tidal ventilation or gas exchange.

## Abstract 2

Slowing the speed of tidal inflations using lower gas flow rates maybe a simple and effective method of reducing preterm lung injury.

## Introduction

The lung is an organ that is in constant motion. Tidal ventilation involves the flow of gas during the mechanical process of inflation and deflation. Most preterm neonates need some form of positive pressure respiratory support after birth. Tidal volume (V_T_) delivery requires a flow of gas to generate pressure and volume change. Despite delivered (or bias) gas flow rates being the fundamental mechanism supporting all positive pressure delivery systems, there are currently no guidelines for setting gas flow rates for neonates (1, 2). Ideally, the bias flow rate should facilitate effective tidal ventilation without imposing excessive work of breathing or injury.

Following birth, the structurally immature preterm lung is at high risk of lung injury (3–5). This is principally due to the mechanical stressors placed upon the lung during the motion of tidal ventilation (6, 7). Injury due to excessive V_T_ (volutrauma), driving pressure (ΔP; barotrauma), and/or inadequate positive end-expiratory pressure (PEEP; atelectasis-mediated injury) are well documented (5, 8). The premise of injury from these mechanisms is based upon an absolute value causing injury. This is overly simplistic as each parameter occurs during continuous motion. The speed of pressure and volume change are likely to be just as important as the absolute amount of change on the fragile preterm lung, whether from direct shearing injury (rheotrauma) or excessive energy transfer (ergotrauma and mechanical power) (9).

The speed of pressure and volume change is determined by the relationship between airway gas flow and lung mechanics (especially resistance). Bias flow rates of 8–15 L/min are often used during neonatal resuscitation, and most modern neonatal ventilators have default flow rates between 8 and 10 L/min. In preterm lambs, flow rates of 8 L/min reduce lung injury compared with 18 and 28 L/min (10). There is a mechanical and lung protective rationale for bias flow rates < 8 L/min in the poorly compliant preterm lung with relatively normal airway resistance and short time constant. In a small study of preterm infants, flow rates of 4 L/min were able to support tidal ventilation (11). Mortality was lower compared with 8 L/min, but the study was underpowered to reliably evaluate major outcomes. To our knowledge, no study has systematically evaluated the role of flow rates < 8 L/min on preterm lung injury.

We hypothesize that the use of low gas flow rates to support positive pressure ventilation (PPV) support of the preterm lung will reduce early injury in the preterm lung. The primary aim of this study was to determine the role of gas flow rates on early lung injury in preterm lambs receiving lung-protective PPV. The secondary aims included assessing the role of gas flow rates on (a) effectiveness of ventilation, mechanical power, parameters of lung motion, and regional ventilation and aeration and on (b) the lung proteome composition.

## Results

Of the 89 lambs studied, Study 1 (15-minute PPV + placental support) included 44 lambs, and Study 2 (90-minute PPV without placental support) included 45 lambs. Three and 4 lambs were excluded in Studies 1 and 2, respectively, due to fetal acidosis (pH < 7.20; *n* = 2), persistent metabolic acidosis during placental support PPV (*n* = 2), or air leak (*n* = 3). The included lambs were well matched (Supplemental Table 1; supplemental material available online with this article; https://doi.org/10.1172/jci.insight.181228DS1).

### Characteristics of tidal lung motion.

**Figure 1:**
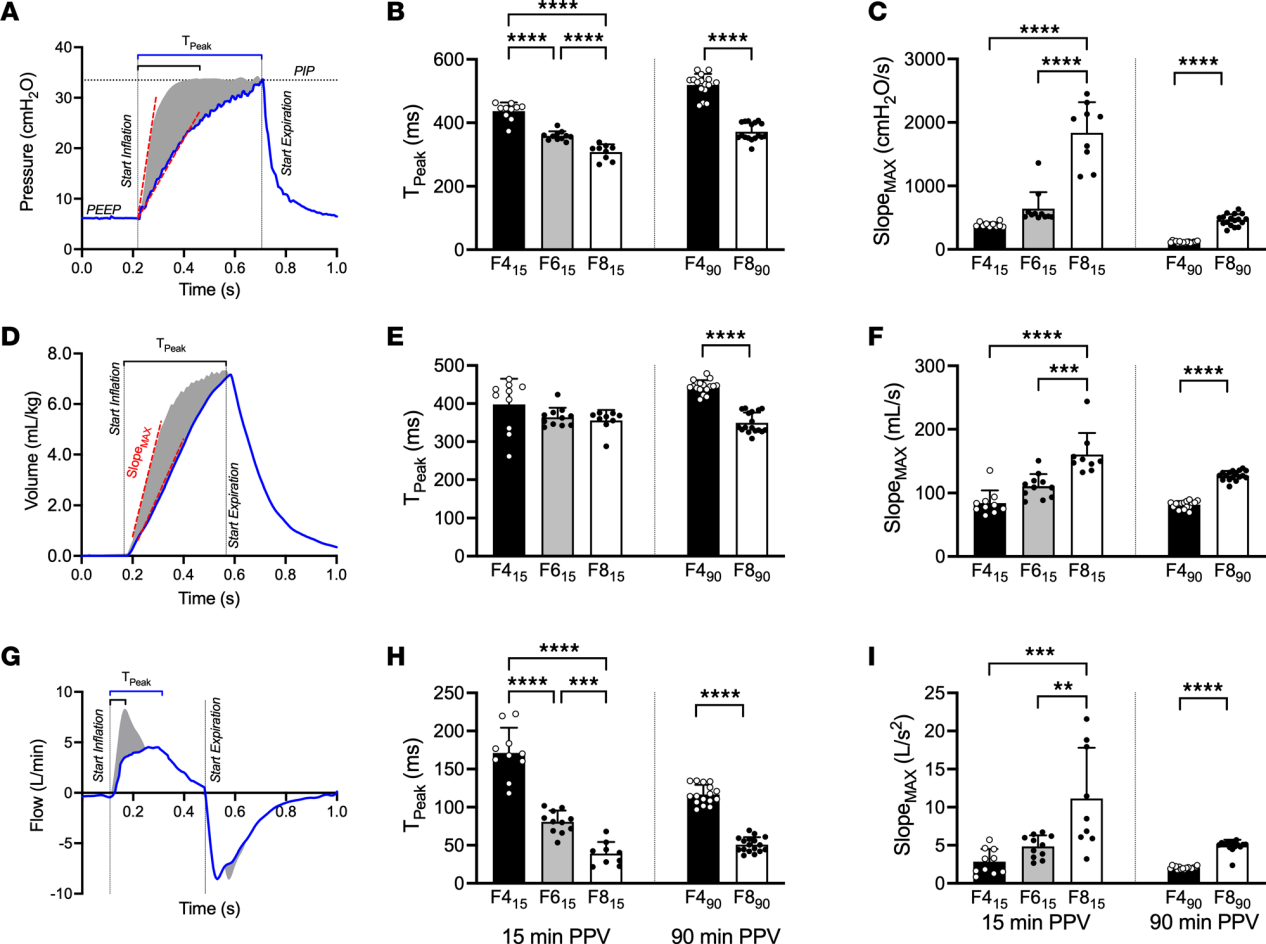
Characteristics of tidal lung motion. (**A**, **D**, and **G**) Representative pressure (**A**), tidal volume (**D**), and flow (**G**) wave during a single respiratory cycle at 4 L/min (blue line) and 8 L/min wave (gray shaded area). (**B**, **E**, and **H**) Time to inspiratory peak (T_Peak_) for pressure (**B**), volume (**E**), and flow (**H**) wave. (**C**, **F**, and **I**) Maximum slope of the inspiratory (Slope_MAX_) pressure (**C**), volume (**F**), and flow (**I**) wave. Slope_MAX_ represents the maximum speed of pressure and volume change, and Slope_MAX_ of flow represents the acceleration of volume in the lung. Black bars represent 4 L/min (F4_15_ and F4_90_) groups, gray bars represent 6 L/min (F6_15_), and white bars represent 8–10 L/min (F8_15_ and F8_90_) bias flow strategy for each ventilation period. Dots represent individual lambs; data are shown as mean ± SD. **P < 0.01, ***P < 0.001, ****P < 0.0001; Tukey’s post hoc test (1-way ANOVA) or *t* test.

Figure 1 summarizes the changes in pressure (Figure 1, A–C), volume (Figure 1, D–F), and gas flow (Figure 1, G–I) during tidal inflations (speed and acceleration of lung motion). The time to peak pressure and flow was slower with decreasing flow rates. Time to peak volume was a mean difference 94 (95% CI; 78, 111) ms (*t* test) slower between F4_90_ and F8_90_. There was no difference in the time to peak volume in the 15 minute PPV groups (*P* = 0.11; 1-way ANOVA). The maximum rate of inflation pressure, volume, and flow change (acceleration of volume) increased with increasing flow rates (all *P* < 0.01). Time at maximum slope of the inflation pressure, volume, and flow wave during inspiration shortened with increasing flow rates (all *P* < 0.01; Supplemental Figure 1). There was no difference in maximum rate of expiratory pressure, volume, or flow change (*P* = 0.11–0.64).

### Lung mechanics, aeration, ventilation, and gas exchange.

**Figure 2:**
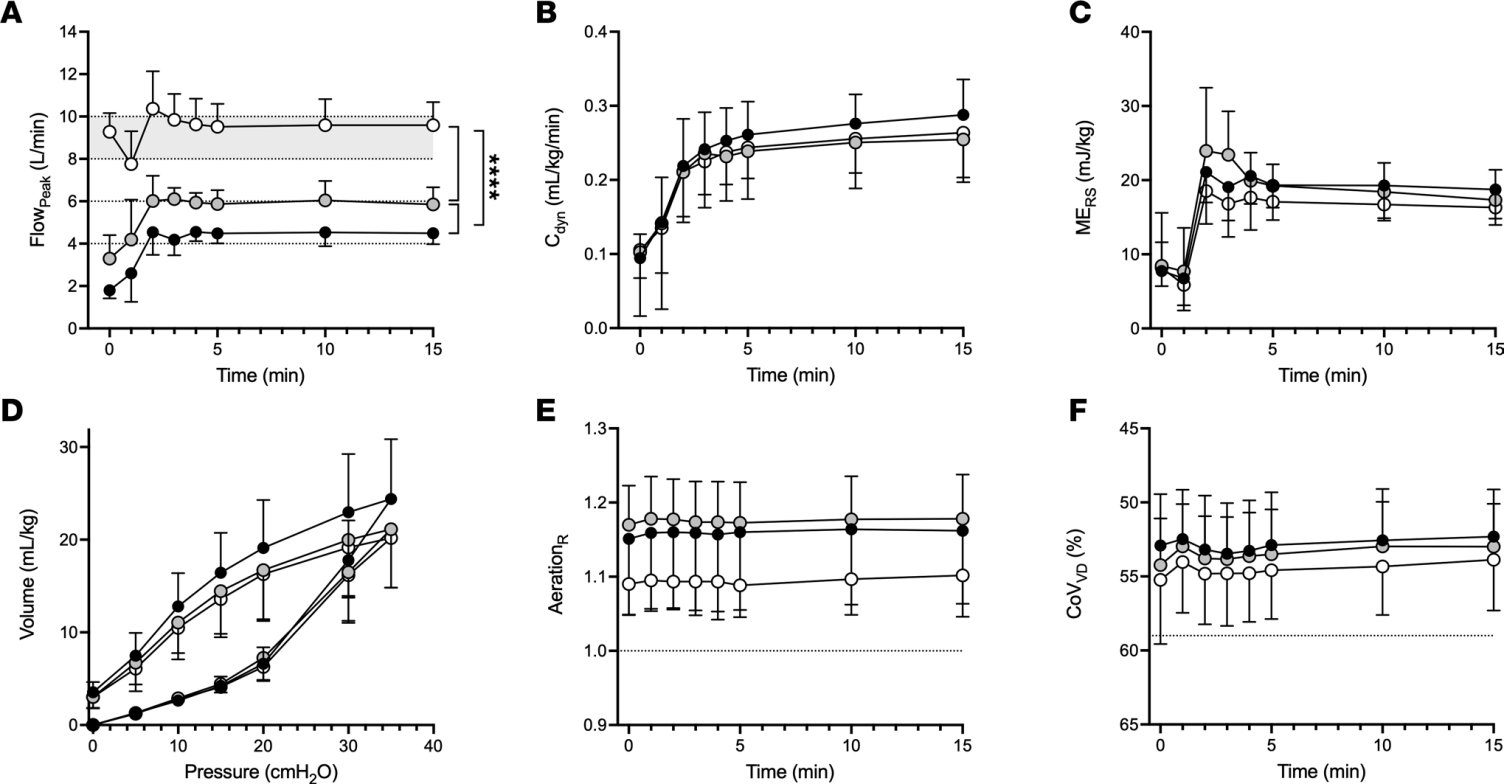
Lung mechanics, aeration, and ventilation following 15 minutes of ventilation using different bias flow rates. (**A**–**F**) Peak inspiratory flow (Flow_Peak_) (**A**), dynamic compliance (C_dyn_) (**B**), mechanical energy of the respiratory system (ME_RS_) (**C**), static pressure-volume curve at 45 minutes (**D**), relative distribution of aeration in the right lung (**E**), and relative gravity dependent distribution of V_T_ along the right to left (CoV_RL_) lung plane (**F**) for the 15-minute ventilation study groups. Relative aeration expressed as the ratio of measured aeration to ideal aeration distribution within the lung. CoV provides a single numerical value (geometric mean) of the gravity-dependent distribution of V_T_ within the chest. Dotted lines indicate the CoV during uniform ventilation. Values less than the value of uniform CoV indicate greater ventilation in the ventral (nondependent) lung compared with the dorsal (dependent) lung accordingly. Black circles represent 4 L/min (F4_15_), gray circles 6 L/min (F6_15_), and white circles 8-10 L/min (F8_15_) bias flow strategy. Data are shown as mean ± SD. ****P < 0.0001; Tukey’s post hoc test (mixed effects model).

**Figure 3:**
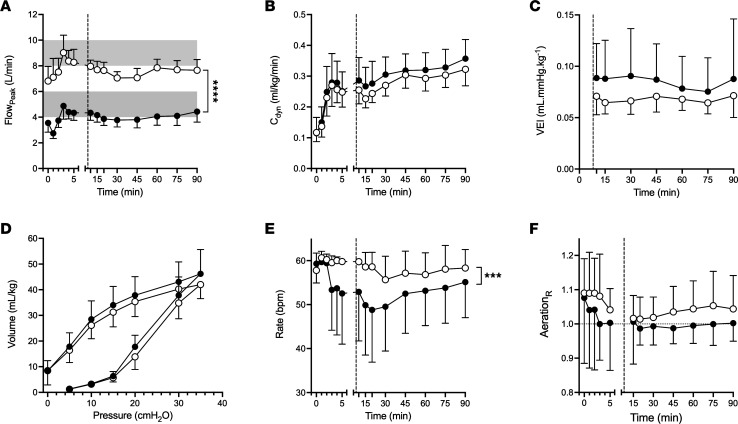
Lung mechanics, aeration, and ventilation following 90 minutes of ventilation using different bias flow rates. (**A**–**F**) Peak inspiratory flow (Flow_Peak_) (**A**), dynamic compliance (C_dyn_) (**B**), VEI (**C**), static pressure-volume curve at 45 minutes (**D**), respiratory rate (**E**), and relative distribution of aeration in the right to left lung plane (**F**) for the 90-minute ventilation study groups. Black circles represent 4 L/min (F4_90_), and white circles 8–10 L/min (F8_90_) bias flow strategy. Data are shown as mean ± SD. Dashed vertical line represents surfactant administration. ***P < 0.001, ****P < 0.0001; Tukey’s post hoc test (mixed effects model).

Figure 2 and Figure 3 describe the lung mechanics for 15- and 90-minute studies, respectively. Maximum peak inspiratory flow approximated allocated target bias flows after completion of initial lung aeration at birth for the 15-minute and 90-minute studies. There was no difference in dynamic respiratory system compliance (C_dyn_), mechanical energy of the respiratory system (ME_RS_), tidal change in pressure (ΔP), V_T_, and tidal mechanical power (MP_tidal_) (Supplemental Figures 2 and 3) between all flow strategies during each study. Change in lung volume (ΔV_L_) from birth was similar during the 15-minute study (Supplemental Figure 2C). Accurate ΔV_L_ from birth could not be calculated in the 90-minute study due to artifact from birth handling at 3 minutes. Total lung capacity was a mean (95% CI) 4.2 (–2.1, 10.5) mL/kg (1-way ANOVA) and 5.2 (–1.3, 11.7) mL/kg (*t* test) different in the F4_15_ and F4_90_ groups compared with respective F8 group (Figure 2D and Figure 3D). In the 90-minute study, the respiratory rate was lower in the F4_90_ group (*P* = 0.0026; mixed effects model; Figure 3E); although mean ventilatory efficiency index (VEI) was higher, the differences were not significant (*P* = 0.53). There was no difference in pH, arterial partial pressure of carbon dioxide (PaCO_2_), base excess, arterial partial pressure of oxygen (PaO_2_), peripheral oxygen saturation (SpO_2_), alveolar arterial difference in oxygen (AaDO_2_), or carotid O_2_ delivery (where applicable) (Supplemental Figure 4).

### Lung aeration and ventilation homogeneity.

Aeration was greater in the right (Figure 2E and Figure 3F) and central lung regions and was relatively under-aerated in the most gravity-dependent (dorsal) lung (Supplemental Figure 5) for all groups. The least gravity-dependent regions were preferentially aerated at 15 minutes (Study 1) but not 90 minutes (Study 2). Overall, aeration was more uniform, but not statistically different, during F8_15_ and F8_90_ than lower rates in all regions (all *P* = 0.20–0.74). Regional ventilation was similar between all groups during each study, with heterogenous ventilation favoring the nongravity-dependent and right lung ventilation (Figure 2F and Supplemental Figure 6). Although F4_15_ had fewer unventilated lung regions during Study 1, and following surfactant in Study 2 (F4_90_), these differences were not statistically significant (Supplemental Figure 6; *P* = 0.10 and 0.33).

### Haemodynamics.

There was no difference in heart rate or carotid artery blood flow between any flow strategies (Supplemental Figure 7).

### Lung morphology and injury.

**Figure 4:**
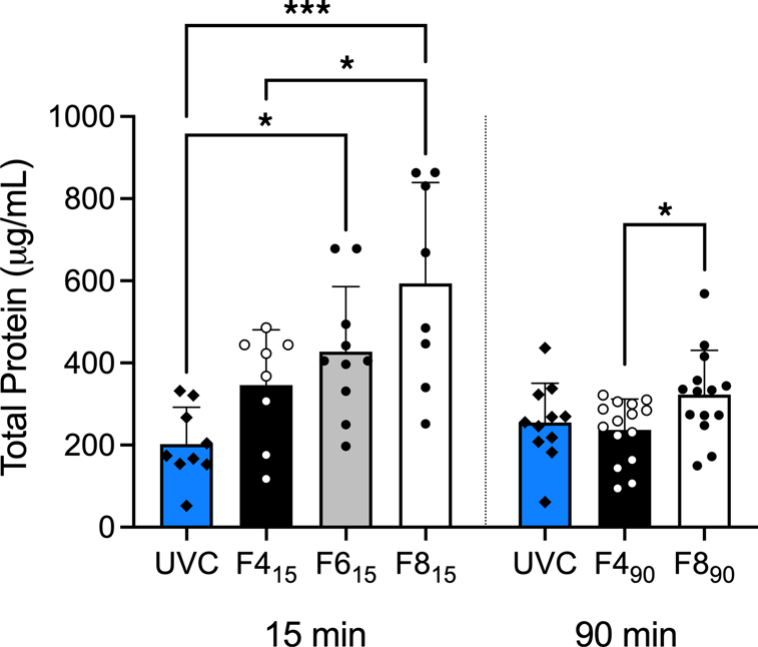
Total protein in the left lung lavage. Blue bars represent unventilated fetal control (UVC) lambs, black bars 4 L/min (F4_15_ and F4_90_) groups, gray bars 6 L/min (F6_15_), and white 8-10 L/min (F8_15_ and F8_90_) bias flow groups for each ventilation period. Dots and diamonds represent individual lambs. All data are shown as mean ± SD. *P < 0.05, ***P < 0.001; 1-way ANOVA with Tukey’s multiple-comparison test.

**Figure 5:**
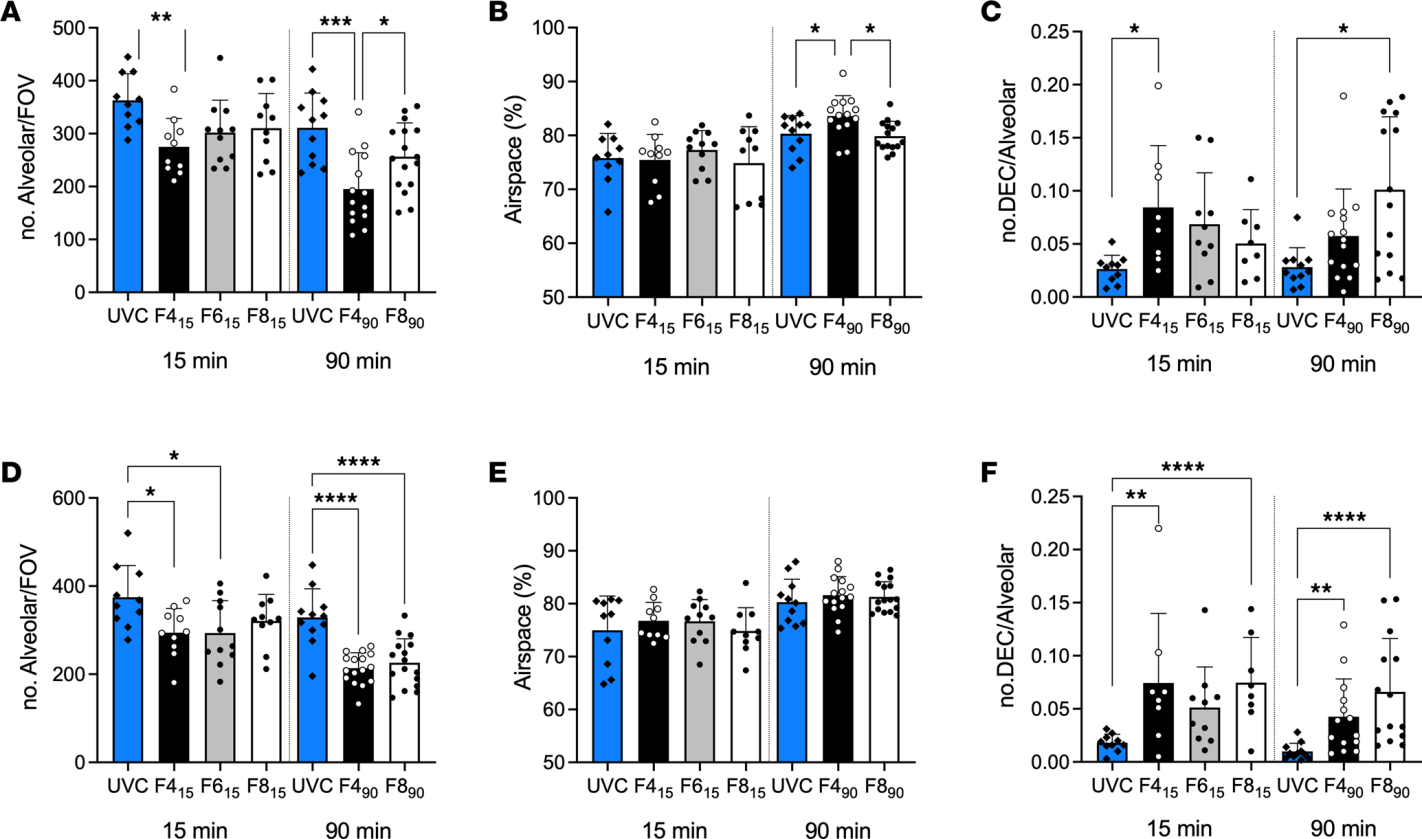
Lung morphology and injury assessment. (**A**–**F**) Number of alveoli per field of view (FOV, lower value more aerated alveoli) (**A** and **D**), percentage of field of view containing airspaces (**B** and **E**), and number of detached epithelial cells per alveoli (**C** and **F**) in the gravity-nondependent (**A**–**C**) and gravity dependent (**D**–**F**) right upper lobe. All analyses were performed from images taken at 10× magnification. Blue bars represent unventilated fetal control (UVC) lambs, black bars 4 L/min (F4_15_ and F4_90_) groups, gray bars 6 L/min (F6_15_), and white 8-10 L/min (F8_15_ and F8_90_) bias flow groups for each ventilation period. Dots and diamonds represent individual lambs. All data are shown as mean ± SD. *P < 0.05, **P < 0.01, ***P < 0.001, ****P < 0.0001; 1-way ANOVA with Tukey’s multiple-comparison test.

Protein concentration of left lung bronchoalveolar lavage fluid (BALF) increased with increasing flow rates at both 15 and 90 minutes (*P* = 0.0003 and 0.047, respectively; 1-way ANOVA), with no difference between an unventilated fetal control group (UVC) and 4 L/min groups (Figure 4). Histological indicators of lung morphology and injury are shown in Figure 5 and Supplemental Figure 8. Overall, the F4_90_ group displayed fewer alveoli per field of view, the most airspace, and fewer detached epithelial cells at 90 minutes.

### Proteome analysis.

**Figure 6:**
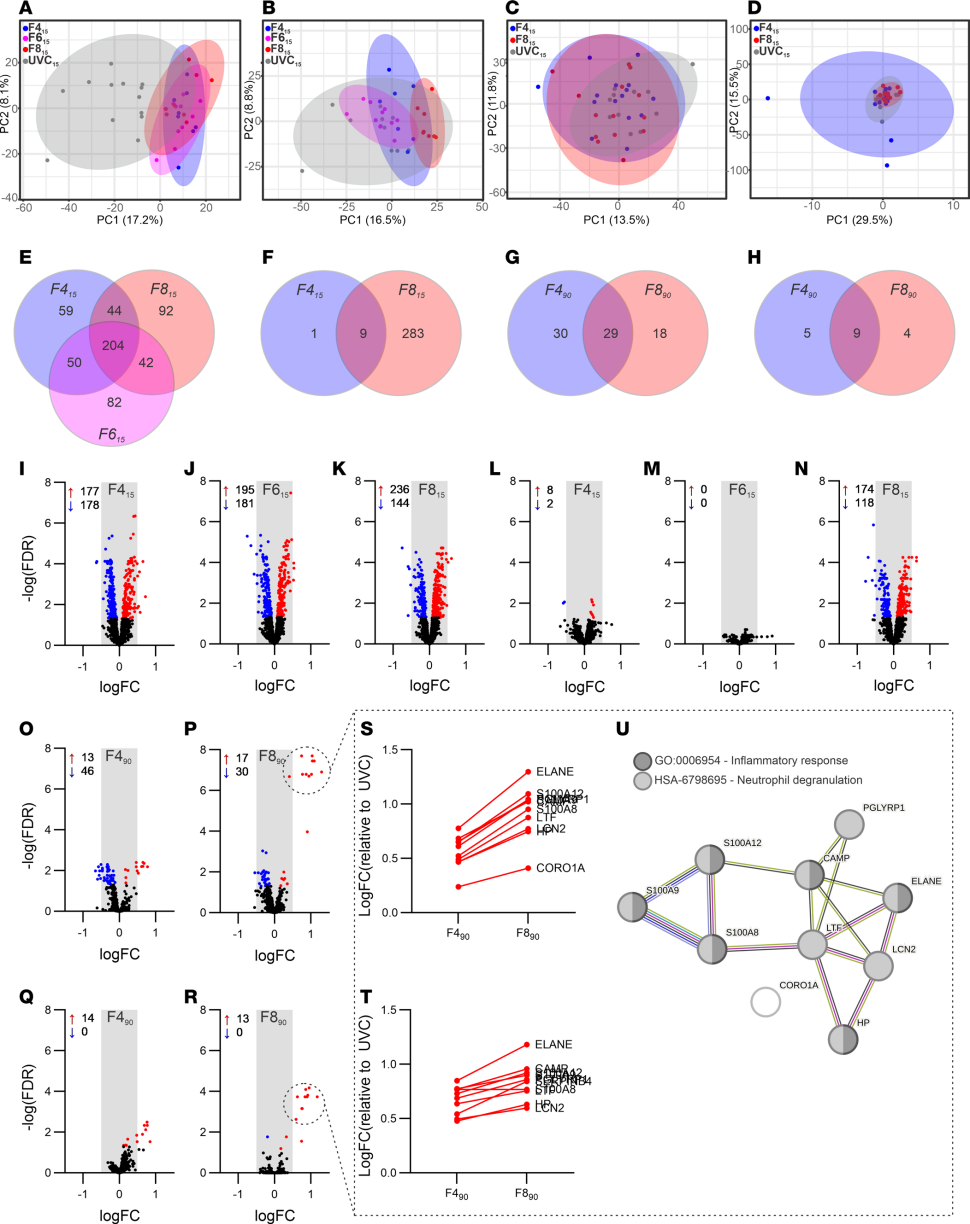
Proteome analysis of nondependent and dependent lung following 15- and 90-minute ventilation at different flow rates. (**A**–**H**) Principal component analysis (PCA) of 15-minute nondependent (**A**), dependent (**B**), and 90-minute nondependent (**C**) and dependent (**D**) lung, with prediction ellipses depicted at a confidence interval of 0.95. Venn diagram of differentially expressed proteins (DEPs) identified in 15-minute nondependent (**E**), dependent (**F**), and 90-minute nondependent (**G**) and dependent (**H**) lung protein datasets. (**I**–**R**) QL F-test with multiplicity correction testing applied using Benjamin-Hochberg method; FDR < 0.05. Volcano plots of 15-minute nondependent (**I**–**K**) and dependent (**L**–**N**) and 90-minute nondependent (**O** and **P**) and dependent (**Q** and **R**) protein data sets, with DEPs that display increased abundance highlighted in red and DEPs with decreased abundance highlighted in blue. (**S**–**U**) Top 10 DEP abundance in 90-minute nondependent (**S**) and dependent (**T**) lung, represented as Log_2_(fold change) relative to UVC_90_, and their protein-protein network interaction STRING (**U**), with high confidence (0.700) and FDR < 0.0001.

In total, 1,543 proteins from Study 1 and 2,588 proteins from Study 2 were included in proteome analysis (Supplemental Tables 2–5). In principal component analysis (PCA), a high degree of separation was apparent between the UVC group and all flow groups in the nongravity-dependent lung at 15 minutes (Figure 6A), while moderate separation was observed in the gravity-dependent lung (Figure 6B). There was no separation between UVC and flow group proteomes in either lung region at 90 minutes (Figure 6, C and D).

Differentially expressed proteins (DEPs) were identified in all ventilation groups (Figure 6, E–R, and Supplemental Table 6), except the F6_15_ gravity-dependent lung, with nongravity-dependent lung regions containing more DEPs compared with gravity-dependent lung regions. DEPs commonly identified between F4_15_, F6_15_, and F8_15_ displayed similar protein abundance (relative to UVC), whereas DEPs at 90 minutes displayed a greater magnitude of change in F8_90_ compared with F4_90_ (Figure 6, S and T). Nine of the top 10 proteins that increased in abundance in F8_90_ compared with F4_90_ were associated with the innate immune response and acute inflammatory response, specifically neutrophil degranulation (Figure 6U). When comparing DEPs between Studies 1 and 2, common proteins were only identified in nongravity-dependent lung proteomes, with RPS3A, RPL10, and CORO1A coidentified between F4_15_ and F4_90_ groups and with CAST, TMA7, NUMA1, and LCP1 coidentified between F8_15_ and F8_90_ groups.

To gain an understanding of protein function and classification, DEPs were analyzed using the PANTHER database. In the nongravity-dependent lung, 83% (Study 1) and 71% (Study 2) of biological processes were commonly identified in all flow groups (Supplemental Figure 9A). The diversity of these processes was comparable in the F8_15_ gravity-dependent lung but reduced in the F4_15_ gravity-dependent lung. This was mirrored in further analysis of cellular processes and protein class characterization (Supplemental Figure 9, B and C). At 90 minutes, differences between F4_90_ and F8_90_ nongravity-dependent and gravity-dependent lung were more obvious.

**Figure 7:**
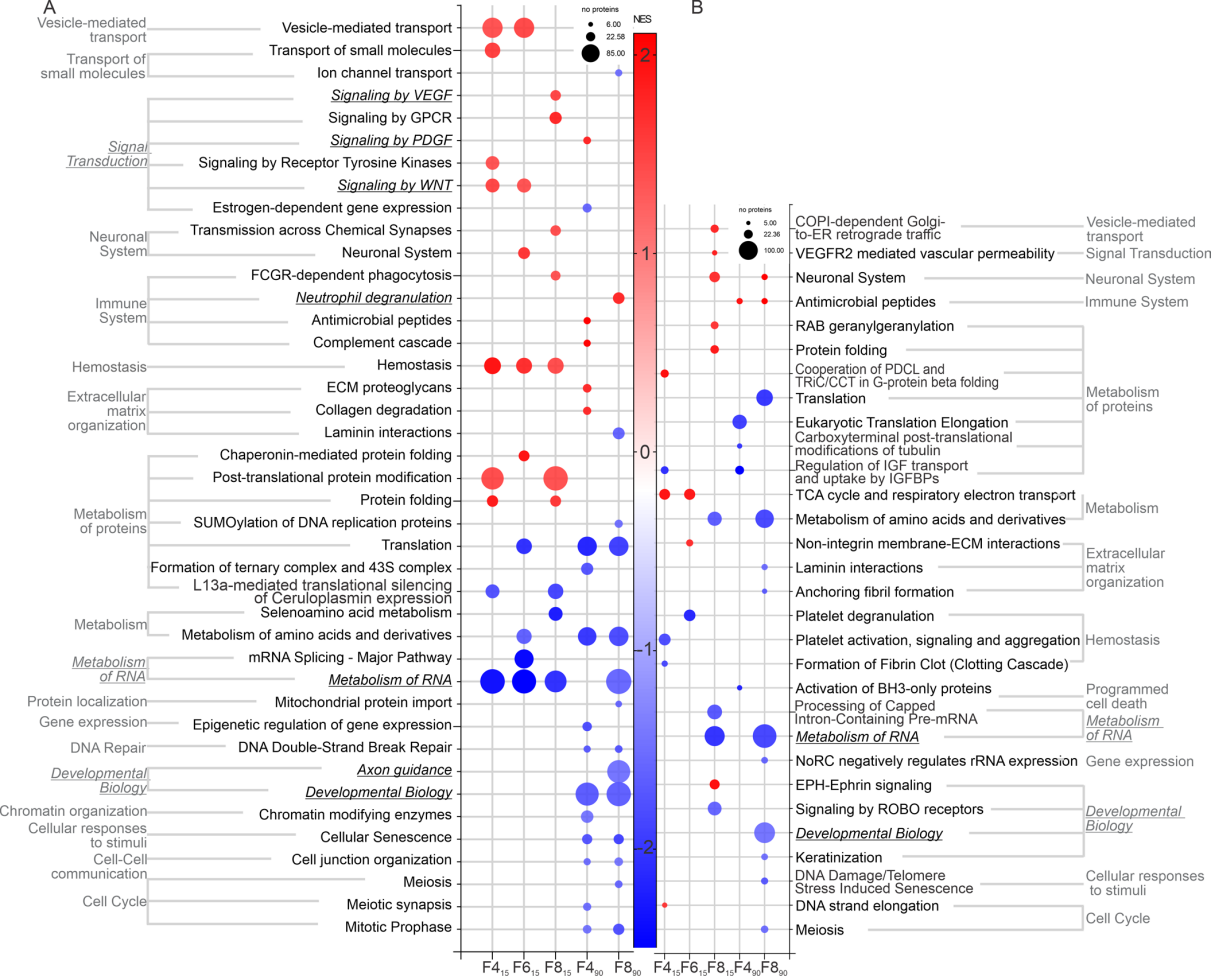
Gene set enrichment analysis of nondependent and dependent lung following 15- and 90-minute ventilation at different flow rates. (**A** and **B**) Reactome pathways identified as enriched (NES > 0; red gradient) or depleted (NES < 0; blue gradient) in gene set enrichment analysis (GSEA) in nondependent (**A**) and dependent (**B**) lung proteomes (FDR < 0.05; weight-set coverage redundancy applied with pathways containing > 5 proteins presented; analysis performed in Webgestalt). Pathways of interest are italicized and underlined. Size of circle indicates number of identified proteins in the pathway. NES, normalized enrichment score.

Gene set enrichment analysis (GSEA) using the Reactome database identified 19 enriched and 22 depleted biological pathways in the nongravity-dependent lung, and 11 enriched and 19 depleted biological pathways in the gravity-dependent lung that were statistically significant (FDR < 0.05; Figure 7). The 8–10 L/min flow had the greatest effect across time and lung regions. In the nongravity-dependent lung, hemostasis and signal transduction pathways were enriched in the flow groups at 15 minutes and decreased developmental biology pathways at 90 minutes, which was less apparent in the gravity-dependent lung. At 90 minutes only, F4_90_ was associated with increased platelet-derived growth factor (PDGF) signaling, which was not seen in the F8 group. Metabolism of RNA was most consistently depleted in flow groups and lung regions.

## Discussion

Preterm birth requires the lung to transition from a fluid-filled to aerated state and then maintain ongoing tidal ventilation despite surfactant-deficiency and structural immaturity (7). Initial lung aeration and subsequent recruitment is actively driven by tidal inflations (4, 12). The neonatal lung does not aerate uniformly, and in preclinical studies, this state of volume heterogeneity is strongly associated with injury (4, 12). Our study aimed to explore the role of tidal lung motion on lung injury, specifically the shear stresses (rheotrauma) caused by the speed of pressure and volume change driven by bias gas flow. We found in our lamb population that supporting the preterm lung with bias flow rates of 4–6 L/min reduced lung injury compared with 8–10 L/min. Most neonatal ventilators, and T-piece resuscitation devices being used in the delivery room, deliver or have recommendation to set bias flows at 8–10 L/min (1, 2). To the best of our knowledge, this is the first time that currently used bias flow rates have been shown to be injurious.

The role of tissue motion in preterm lung injury has been inadequately studied, limited to a single lamb study and small clinical human study (10, 11). In contrast, vascular shear stress and resultant injury is well described (13, 14). There is a strong rationale for flow-related lung injury in the preterm lung. The preterm respiratory system has a low resistance and compliance. This scenario places few brakes on rapid lung motion once enough inflation pressure is applied during both spontaneous breathing and PPV (3). Furthermore, the preterm lung is not yet adapted to the movement of air or the flow rates required to support gas exchange. Preterm lung injury is generally defined by the effect of the absolute magnitude of a parameter, such as V_T_, inflating pressures, PEEP, and V_L_ (5, 6). In our study, we applied a standardized ventilation strategy that conformed with the current concepts to minimize injury from these mechanisms in preterm lambs: avoidance of excessive V_T_, inflating pressure and atelectasis (dynamic PEEP at birth followed by 8 cmH_2_O PEEP), antenatal steroid exposure, and surfactant treatment (Study 2) (3, 4, 15–17). Bias flow was the only ventilator-derived parameter that differed between groups, suggesting that the increase in total protein concentration and different alveolar morphology observed at 15 and 90 minutes was due to the independent effect of the higher flow rates.

As the duration of PPV increased, the F8_90_ group demonstrated increased alteration in the immune response and acute inflammatory proteome compared with F4_90_. This likely reflects an acute injurious effect from the higher flow rates, as demonstrated by the increase in detached epithelial cells. This mechanotransducive injury from flow is likely multifactorial and includes tissue shear stress, acceleration, different flow types, energy transference, and direct pressure and volume effects. Our study could not delineate the mechanism of injury, but the different processes and protein classes seen throughout lung regions, and similar ME_RS_ values, suggest that considering rheotrauma as simply shear stress or force-related energy transfer may be too simplistic. Interestingly, F4_90_ was associated with increased PDGF signaling, which is important for alveolarization, with defective signaling being a primary feature of bronchopulmonary dysplasia in humans (18, 19). It is possible that the slower flow rates allow alveolar cells to adapt better to the increased mechanical tensions associated with ventilation after birth (20).

There was greater differentiation in the proteome for all flow rates against UVC in the nongravity-dependent lung. This is to be expected, as aeration and ventilation patterns favored the nondependent regions. The differences in protein classes, processes, and pathways between gravity-dependent lung regions further illustrate the need to define preterm lung injury based on regional heterogeneity. Unfortunately, the differences in right and left injury patterns could not be assessed. Although there were differences in lung morphology between UVC and flow groups, the findings were more subtle than other studies with smaller sample sizes (21–23) and likely reflect the more lung protective approach to ventilation in our study.

Lower-bias flow rates did not negatively affect lung mechanics or gas exchange. This is important and supports the need for clinical investigation of lower-bias flow rates and, importantly, slower rates of pressure and volume changes during invasive support. A previous study from 1968 of different gas flow rates in 9 neonates supported using a slow rate and long inspiratory time (Ti) approach for oxygenation, but this approach is now considered injurious (6, 24). The similar VEI between the F4_90_ and F8_90_ groups was achieved due to the lower respiratory rate in the F4_90_ group, suggesting a similar response to that reported by Bach and colleagues in term lambs (25). Importantly, respiratory rate was titrated to maintain the PaCO_2_ target and Ti fixed. This indicates that CO_2_ clearance was improved in the F4_90_ group likely related to less lung injury. We did not measure the effect of flow rates on ventilation-perfusion matching. This may be affected by the lower mean airway pressure resulting from the longer pressure rise time during inflation at slower flow rates and should be considered in future studies.

Many modern ventilators do not allow direct titration of bias flow. Rather, the clinician can alter the pressure rise time. In experienced hands, arguably this is a more clinically usable approach. Physiologically, the Ti should first be set to the time constant of the lung (compliance × resistance) and titrated to the flow wave shape (26). Thereafter, our data suggest that the rise time can be slowed to obtain end-inspiratory pressure near the end of Ti. Such an approach will account for the variability in optimal flow rates due to a difference in lung maturation (gestation) and mechanics (disease severity) (24). Whether lower flow rates can be tolerated during noninvasive ventilation cannot be concluded from this study. We did not measure work of breathing in our study, and increased work of breathing may generate rapid inspiratory flows. Studies in neonates of bias flow during noninvasive ventilation need to include measures of work of breathing (such as esophageal manometry). Given the simplicity of altering bias flow during noninvasive support and the lack of guidance for clinicians (1, 2), there is a clear need to understand its role in preterm injury.

In our study, we used a continuous bias flow ventilator, a mechanism of pressure delivery similar to T-piece devices used in the delivery room. Peak inspiratory flow rates approximated but were not always equal to the set bias flow. This is not surprising; although bias flow and inspiratory flow are nearly linear (25), pressure gradient drops through the circuit and endotracheal tube, laminar and turbulent flow patterns through the airway, changing respiratory mechanics, and the mechanism of titrating bias flow in the SLE5000 will all alter flow rates (27). This is especially so during the birth transition, when all these factors are likely to be greater as the lung transitions from a high-resistance fluid to low-resistance aerated state (4, 12, 28).

There are additional limitations to our study, some of which we have reported in detail before, including those related to EIT and proteomics (3, 4, 29–31). There were more female lambs in Study 2 relative to male lambs, and the opposite was true in Study 1. This may have influenced the injury outcomes and could, in part, explain the higher protein concentrations in Study 1 despite the shorter ventilation period. However, the lambs in Study 1 were also more immature and did not receive surfactant. The study was not powered to subgroup analysis by sex. Unlike human neonates, the lambs were apnoeic and intubated. This approach, though well established (32), limits translation to the clinical setting and was intentional. Spontaneous breathing generates negative flow inspiratory gradients, which in themselves may generate injury, especially in the delivery room where spontaneous inflations may not be synchronous with PPV-mediated inflations. This would create very complex flow patterns involving variable turbulent and laminar flow states. Our aim was to understand both the initiation and potentiation of early lung injury, necessitating the established but nonclinical prolonged placental support model in Study 1 (21). Allowing spontaneous breathing during placental support while maintaining ewe and fetal analgesia is technically and ethically difficult. This justifies why we chose different gestational age ranges. Study 1 was embedded in a larger program in 124-day to 127-day gestation lambs also differentiating the roles of inflating pressure and V_T_ on initiating lung injury (3, 33), but these lambs were at a gestation that is harder to achieve spontaneous breathing at birth (34). We also wanted to understand the independence of flow-mediated lung injury. Now that the role of PPV is better understood, the role of breathing can be interrogated during different permutations of noninvasive and invasive support with synchronous and asynchronous breathing in the future.

### Conclusions.

Set bias flow and resultant speed of pressure and volume change in the lung during tidal ventilation are independent mechanisms of neonatal lung injury that have been largely underrecognized. This study demonstrates that supporting the preterm lung from birth with slower flow rates than currently used clinically may result in less lung injury without compromising tidal ventilation or gas exchange. If replicated in human studies, implementing lower gas flow rates into neonatal care maybe a simple and cost-effective method of lung protection.

## Methods

Supplemental Methods are available online with this article.

### Sex as a biological variable.

For this study, both male and female preterm Border Leicester cross lambs were studied. Randomization to allocated flow strategy occurred before birth, and sex was not known at randomization. Similar findings are reported for both sexes. Sex may have been a variable, but power to sex as a variable was not possible for reasons of ethical reduction.

**Figure 8:**
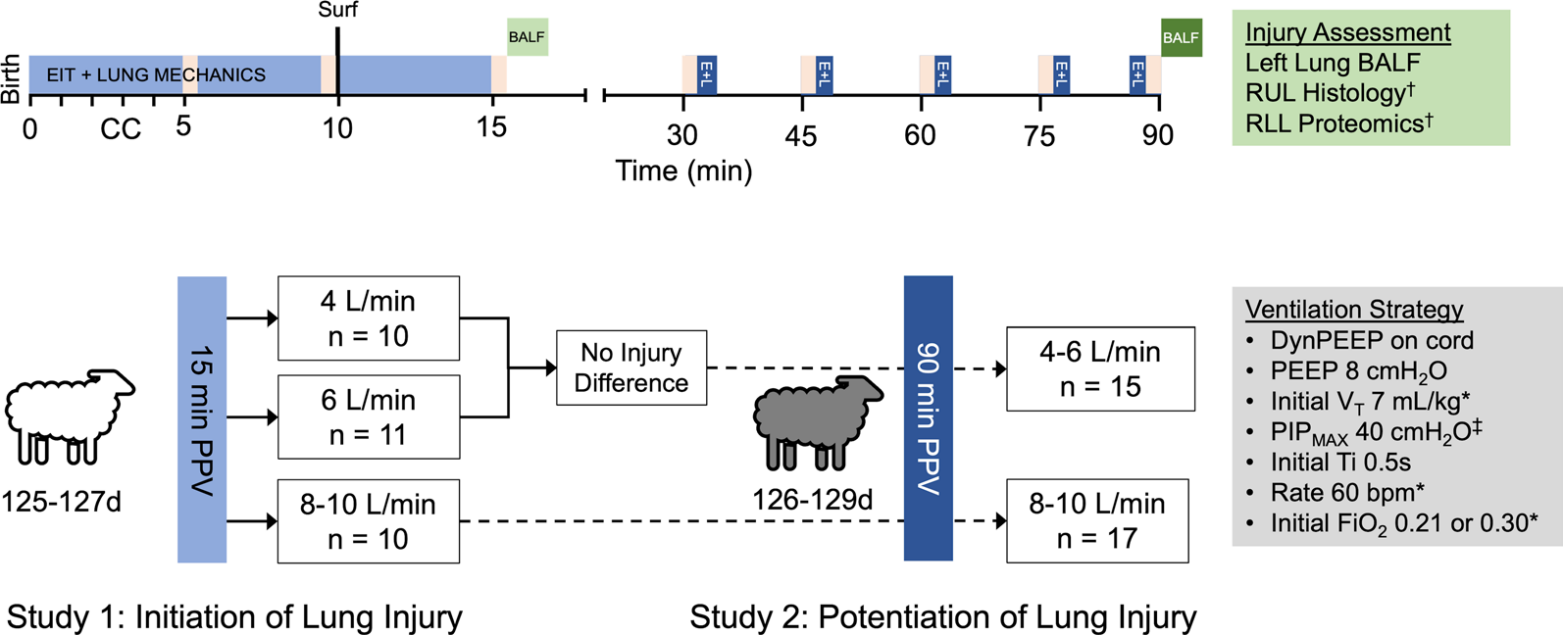
Summary of study design. Light blue represents methods common to both study phases. Dark blue and green represent methods limited to the 90-minute positive pressure ventilation (PPV) study (Study 2). *Tidal volume (V_T_) and PPV rate were adjusted based on arterial blood gas analysis (pink shaded boxes). Fraction of inspired oxygen (FiO_2_) remained 0.21 throughout Study 1 and was commenced at 0.30 and adjusted to maintain preductal peripheral oxygen saturations 91%–95% in Study 2 after the umbilical cord was cut. †Right upper (RUL) and lower (RLL) lobe histology and proteomics performed on the gravity dependent and nondependent regions of each lobe. ‡Maximum inflation pressure (PIP_MAX_) was set at 40 cmH_2_O, but transient increases to 50 cmH_2_O were permitted during the initial dynamic PEEP maneuver (DynPEEP) at birth to facilitate aeration if needed (4, 16).

Some aspects of the methodology have been reported previously (3, 4, 35, 36). To address the study aims, 2 consecutive studies were performed (Figure 8). The first aimed to determine the role of bias flow rates on initiating early lung injury pathways at birth (Study 1). For Study 1, 12-day to 127-day preterm Border Leicester cross lambs (term = approximately 145 days) were studied during 15 minutes of PPV while on placental support using 4 (F4_15_), 6 (F6_15_) ,and 8–10 (F8_15_) L/min flow (*n* = 10–11/group). Lambs received apnoeic oxygenation and perfusion on placental support for 30 minutes to allow expression of injury markers (3, 21, 33).

Study 2 investigated the role of 4–6 (F4_90_) versus 8–10 (F8_90_) L/min flow (*n* = 15–17/group) on potentiation of lung injury during 90 minutes of PPV without placental support in 126-day to 129-day lambs. The flow rates and group sizes for Study 2 were informed by the results of Study 1 to ensure likelihood of treatment difference and maximize animal reduction. Group allocation was randomly applied in both studies. In total, 11–13 lambs per study were also studied as unventilated controls (UVC) for injury comparisons.

### Experimental instrumentation.

All lambs were born via cesarean section to glucocorticoid-treated ewes and intubated and instrumented as described previously (3, 37). Following full exteriorization, all lambs were ventilated supine with active warming. Anesthesia and analgesia were maintained with ketamine and midazolam infusions.

### Measurements.

Heart rate, carotid artery pressure and flow, airway pressure, gas flow, and V_T_ at the airway opening (Florian, Acutronic Medical Systems) were measured continuously from birth. Global and regional V_L_ changes were acquired by electrical impedance tomography (EIT; Pioneer System, Sentec) at 48 scans/s (37). Arterial blood analysis was performed at 5 minutes and then every 15 minutes from ventilation onset.

### Ventilation strategies and general management after birth.

Except for allocated bias flow, a common PPV strategy (SLE5000, SLE Ltd.) was employed in accordance with current lung protective concepts summarized in Figure 1. Lambs in Study 2 received the initial 3 minutes of PPV with placental support to mimic deferred cord clamping. Ti was commenced at 0.5 seconds and shortened if end-inspiratory 0 L/min flow was > 20% of Ti. Lambs in Study 2 received 240 mg of porcine surfactant (Curosurf, Chiesi) at 10 minutes. At the end of assigned experimental period, a BAL was performed and the static in vivo pressure-volume (PV) curve generated from atmosphere to 35 cmH_2_O to determine static lung mechanics (3, 4). Then a lethal dose of pentobarbitone was administered (35–39).

### Data acquisition and analysis.

EIT data, ΔP, V_T_, C_dyn_, and minimum (diastolic), maximum (systolic), and average carotid blood flow were calculated at key time points (3, 4, 16, 40). Dynamic MP_tidal_ (41), ME_RS_ (42), and VEI were calculated post hoc (see Supplemental Methods for formulas) (23, 25, 43). Time-course EIT image data were reconstructed using an anatomically correct custom-built lamb algorithm (31, 36, 44) filtered to the respiratory domain (IBEX software package, Sentec) (37). ΔV_L_ from the preaerated state was calibrated from the static PV curve (35, 39). Relative aeration (to anatomical size) (36, 37, 39) and center of ventilation (CoV) within the gravity-dependent and -nondependent regions and within right and left lungs were calculated (31).

Protein concentration of left lung BALF were determined using the Lowry method (45). Histology of the dependent and nondependent zones of the right upper lobe (fixed at 20 cmH_2_O) were assessed using our previously described standardized criteria in ImageJ (NIH) as detailed in the Supplemental Methods (29, 36, 39, 46).

Lung tissue samples were collected from the gravity-dependent and -nondependent zones of the right lower lobe for quantitative mass spectrometry-based proteomics using our previously detailed methods (3, 33). Comparisons between ventilated groups versus study-specific UVC groups were performed using the EdgeR Package (RStudio) to identify DEPs, with significance set to FDR < 0.05 (47, 48). GSEA was performed in Webgestalt to identify altered biological pathways within the Reactome database, with FDR < 0.05 (49), and PANTHER was used to characterize biological processes and protein classes of DEPs (50).

### Statistics.

Eight to 10 lambs/group were previously able to demonstrate differences in our histological markers following 15 minutes of PPV (3, 51), and 15–20 lambs/group following 90-minute PPV at each allocated gestation (80% type 1 error, *P* = 0.05) (4). All nonproteomics data were investigated with either 2-tailed *t* tests, 1-way ANOVA, or mixed effects analysis (ventilation strategy and time as variables) and an appropriate post hoc test analysis. A *P* value less than 0.05 was considered significant.

### Study approval.

All techniques and procedures were approved by the Animal Ethics Committee of the Murdoch Children’s Research Institute, Melbourne, Australia (4 May 2020; A923, 23 September 2021; A943, 3 August 2022; A956) in accordance with National Health and Medical Research Council guidelines (Australia). The ARRIVE Statement for this study is available at Tingay, David (2022): University of Melbourne https://doi.org/10.26188/25242616.v1

### Data availability.

All data, including raw data used for all figures and analysis, and allocation of specific subjects in previously published material is available upon request to the corresponding author from 3 months following article publication to researchers who provide a methodologically sound proposal, with approval by an independent review committee (“learned intermediary”). Proposals should be directed to david.tingay@mcri.edu.au to gain access. Data requestors will need to sign a data access or material transfer agreement approved by MCRI. The ARRIVE Statement for this study and all the proteome data sets used for analysis are available at Tingay, David (2024): University of Melbourne. https://doi.org/10.26188/25242616.v1 and https://doi.org/10.26188/25242589 The mass spectrometry proteomics data have been deposited to the ProteomeXchange Consortium (http://proteomecentral.proteomexchange.org) via the PRIDE partner repository with the data set identifier PXD041917 (Study 1) and PXD050305 (Study 2). Value for all data points in graphs are reported in the Supporting Data Values file.

## Author contributions

DGT and PMPF developed the concept, designed the experiment, and interpreted the data. DGT, PMPF, JC, KK, ED, AS, SID, MS, and MF were involved in lamb experimental work. QHP, DWG, HF, MF, and PMPF performed proteomic experiments, and MF, TKQ, and PMPF performed the injury and proteomics analysis. DGT supervised all aspects of the study and subsequent data analysis. JC, KK, SID, ED, and DGT performed the ventilator waveform signal and EIT analysis. All authors participated in data interpretation under supervision of DGT and PMPF. DGT wrote the first draft, and all authors contributed to redrafting the manuscript.

## Supplementary Material

Supplemental data

Supplemental tables 2-6

Supporting data values

## Version 1 08/06/2024

In-Press Preview

## Version 2 09/24/2024

Electronic publication
