# Supplementary material for: Speed of lung inflation at birth influences the initiation of lung injury in preterm lambs
Source: JCI Insight. 2024 Aug 6;9(18):e181228. doi: 10.1172/jci.insight.181228 (PMC11457856; doi:10.1172/jci.insight.181228)
Supplement: Supplemental data [file jciinsight-9-181228-s143.pdf]

Low gas flow rates at birth influence the initiation of lung injury in preterm lambs

David G Tingay[1,2], Monique Fatmous[1], Kelly Kenna[1], Jack Chapman[1,2], Ellen Douglas[1], Arun Sett[1,3,4], Qi Hui Poh[1], Sophia I Dahm[1], Tuyen Kim Quach[1,2], Magdy Sourial[1,5], Haoyun Fang[6,7], David W. Greening[6,7,8], Prue M Pereira-Fantini[1,2]

[1]Neonatal Research, Murdoch Children's Research Institute, Parkville, Australia

[2]Department of Paediatrics, University of Melbourne, Melbourne, Australia

[3]Newborn Services, Joan Kirner Women's and Children's, Sunshine Hospital, Western Health, St Albans, Australia

[4]Department of Obstetrics and Gynaecology, University of Melbourne, Melbourne, Australia

[5]Translational Research Unit, Murdoch Children's Research Institute, Parkville, Australia

[6]Molecular Proteomics, Baker Heart and Diabetes Institute, Melbourne, Australia

[7]Baker Department of Cardiometabolic Health, The University of Melbourne, Melbourne, Australia

[8]Baker Department of Cardiovascular Research, Translation and Implementation, La Trobe University, Melbourne, Australia

1. **Detailed Methodology**
2. **Results:**
    a. **Online Supplementary Table E1. Subject characteristics**
    b. **Online Supplementary Figure E1. Additional characteristics of tidal motion**
    c. **Online Supplementary Figure E2. Additional lung mechanics (15 min PPV study Group)**
    d. **Online Supplementary Figure E3. Additional lung mechanics (90 min PPV study Group)**
    e. **Online Supplementary Figure E4. Gas exchange data**
    f. **Online Supplementary Figure E5. Ventral-Dorsal (gravity-dependent) aeration**

g. **Online Supplementary Figure E6. Additional regional ventilation data**

h. **Online Supplementary Figure E7. Cardiovascular results**

i. **Online Supplementary Figure E8. Additional histology data**

j. **Online Supplementary Figure E9. Characterisation of differentially expressed proteins of non-dependent and dependent lung following 15 and 90 min ventilation at different bias flow rates.**

Online Supplementary Tables 2-6 contain all the proteome datasets used for analysis for all studies and are available as raw data (csv format) at Tingay, David (2024): University of Melbourne: https://doi.org/10.26188/25242589

Supplementary Table 6 contains a list of all differentially expressed proteins identified in treatment groups in Study 1 and Study 2.

DETAILED METHODOLOGY

Sex as a biological variable

For this study, both male and female preterm Border-Leicester cross lambs were studied. Randomisation to allocated flow strategy occurred before birth and sex was not known at randomisation. Similar findings are reported for both sexes. Sex may have been a variable but power to sex as a variable was not possible for reasons of ethically reduction.

All techniques and procedures were approved by the Animal Ethics Committee of the Murdoch Children's Research Institute, Melbourne, Australia (4 May 2020; A923, 23 September 2021; A943, 3 August 2022; A956) in accordance with National Health and Medical Research Council guidelines (Australia). The ARRIVE Statement for this study is available at Tingay, David (2022): University of Melbourne https://doi.org/10.26188/25242616.v1 Some aspects of the methodology have been reported in detail previously.(1-4)

Experimental Instrumentation

To address the study aims, two interconnected studies were performed and are summarised in the main manuscript **Figure 1**.

Study 1 aimed to determine the role of gas flow rates on initiating early lung injury pathways at birth: Surfactant-deficient 124-127d preterm Border-Leicester cross lambs (term ~145d) were studied. All were born via caesarean section under general anaesthesia to ewes that received 11.7 mg of betamethasone 24 and 48 hours prior to delivery.(3) The head and chest of the lamb was exteriorised and instrumented. This included insertion of a carotid arterial and external jugular line, placement of a 3 mm flow probe around the contralateral carotid artery (Transonic, AD Instruments, Sydney, Australia), intubation (4.0 cuffed endotracheal tube) and applying a custom-built electrical impedance tomography (EIT) belt around the chest as described previously.(5, 6) Lambs were then fully

exteriorised, dried and placed supine on heat pads on the ewes' abdomen under heat lamp with placental support maintained. 4 mm and 6 mm flow probes were placed around an umbilical artery and vein, respectively, as part of another study. Lung liquid was passively drained before commencing respiratory support. Temperature was monitored via a rectal thermistor and external heating adjusted accordingly. Anaesthesia and analgesia were maintained throughout the study period with ketamine and midazolam infusions to supress spontaneous breathing.

Study 2 investigated the role of gas flow rates on potentiation of lung injury during 90 min of positive pressure ventilation (PPV) without placental support in 126-129d lambs. In the interest of animal reduction the data from Study 1 was analysed before Study 2 commenced. There was no difference in total protein and mechanics outcomes from 4 and 6 L/min. The two gas flow intervention groups were determined to be 4-6 versus 8-10 L/min. Surfactant-deficient 126-129d preterm Border-Leicester cross lambs (term ~145d) were studied. All were born via caesarean section under general anaesthesia to ewes that received 11.7 mg of betamethasone 24 and 48 hours prior to delivery. All lambs were instrumented as per Study 1 during placental support. After instrumentation, lambs received the first 3 min of PPV with placental support to facilitate lung aeration (deferred cord clamping).(7, 8) At 3 min the umbilical cord was cut, the lamb weighed (with ongoing PPV) and the lamb placed supine in an open resuscitaire with overhead heating and a heat mat for the remainder of the experimental period. Anaesthesia and analgesia were maintained throughout the study period with ketamine and midazolam infusions to supress spontaneous breathing.

Lambs from each Study were breed from the same ewe flock (MCRI sole supplier) who were exposed to the same environmental conditions.

Measurements

Heart rate, carotid artery and umbilical vessel flow (Study 1 only), airway pressure, gas flow and tidal volume (V_T) at the airway opening (Florian, Acutronic Medical Systems AG, Hirzel, Switzerland) were measured continuously from birth. In addition, arterial blood pressure and pre-ductal peripheral oxygen saturation (SpO_2) were measured continuously in Study 2. Global and regional lung volume changes were acquired by EIT (Pioneer System, Sentec AG, Landquart, Switzerland) at 48 scans/s.(5, 6, 9) Arterial blood analysis was performed at 5 min and then every 15 min from birth.

Randomisation process

Lambs were randomly assigned to a ventilation strategy before delivery in a two-step process. Initial randomisation occurred when obstetric ultrasound scans were performed at the farm (usually 1-2 months before delivery of ewes to the research facility) to facilitate even allocation of lambs across groups by gestational age (days), singleton and twin pregnancy and delivery order if a twin pregnancy. As sex could not be predicted before delivery, reallocation was allowed (where possible) before any fetal instrumentation if intra-group imbalances in sex were identified, and only after half of the allocated sample size for each group was studied. In the case of multiple parity ewes, no lambs were assigned to the same intervention from the same mother. Further, lambs with severe growth restriction and/or severe fetal acidosis (defined as a cord pH <7.20) were excluded due to the independent impact each may have on lung injury and need for larger sample sizes (principal of reduction). Where possible lambs were replaced with the same group.

Ventilation Strategies

Lambs in Study 1 were randomly allocated to receive PPV using 4, 6 or 8-10 L/min bias flow. Lambs in Study 2 were randomly allocated to 4-6 L/min or 8-10 L/min. PPV was applied with the SLE5000 infant ventilator (SLE Ltd, South Croydon, UK). The SLE5000 is a continuous bias flow mechanical ventilator (default 8-10 L/min) but the clinician does not directly set bias flow in L/min. Rather, the clinician alters delivered bias flow by changing the rise time (inflation slope) setting. To determine

the allocated bias flow rates, the rise time settings were calibrated to those achieving a flow of 4 and 6 L/min using an infant test lung set at a respiratory system compliance (C_{RS}) of 1 ml/cmH_2O. The default bias flow of the SLE5000 of 8-10 L/min was also confirmed using this calibration.

Except for allocated flow rates, a common PPV strategy was employed in accordance with current lung protective concepts (summarised in Figure 1):

1) **Initial Settings at birth:** Inspiratory time (Ti) 0.5 s, Volume targeted ventilation (VTV) at 7 mL/kg tidal volume (V_T) and maximum positive inspiratory pressure (PIP) 40 cmH_2O, positive end-expiratory pressure (PEEP) 8 cmH_2O and rate 60 inflations per minute. Lambs in Study 1 were supported in 0.21 FiO_2 throughout and Study 2 commenced PPV in 0.30 FiO_2.
2) **Initial Dynamic PEEP aeration manoeuvre (0-3 min):** A step-wise PEEP strategy from an initial 8 cmH_2O PEEP.(3) PEEP was manually increased by 2 cmH_2O every 10s until 20 cmH_2O (P_{MAX}), and then decreased step-wise to 8 cmH_2O prior to a transient re-recruitment at 20 cmH_2O for 10s (total duration approximately 150-180s).(1, 10)
3) **Ongoing PPV settings:** PPV continued at 8 cmH_2O for the remainder of the allocated ventilation period (15 min Study 1, 90 min Study 2). The Ti could be shortened if more than 20% of the flow wave was at 0 L/min at end-inflation. The V_T was adjusted to maintain an arterial partial pressure of carbon dioxide ($PaCO_2$) of 45-60 mmHg. In the case of a low $PaCO_2$. first the V_T was weaned using a stepwise standardised protocol until 5.5 mL/kg. There after ventilator rate was weaned. For Study 2, FiO_2 was adjusted to maintain SpO_2 91-95% after the umbilical cord was cut.

General management after birth

Lambs in Study 1 were supported with their assigned strategy until 15 min. Lambs were then maintained on placental support and external heat for 30 min to allow upregulation of injury markers as per the method of Hillman and co-workers and our previous study.(11-13) At 45 min,

bronchoalveolar lavage was repeated, a static *in vivo* pressure-volume curve was generated from atmosphere to 35 cmH_2O to calibrate the EIT signal and determine static lung mechanics and all lambs received a lethal dose of pentobarbitone.(3-5, 14, 15)

Lambs in Study 2 received 240 mg of porcine surfactant (Curosurf, Chiesi, Parma, Italy) at 10 min, slowly administered over 30-60 s via a closed suction connector (to maintain PPV) and 8 FG catheter pre-measured to the ETT tip.(1) At 90 min, a bronchoalveolar lavage was performed and the static *in vivo* pressure-volume curve generated as per Study 1. All lambs received a lethal dose of pentobarbitone at the end of the study.

11-13 age matched fetal lambs per study received a lethal dose of pentobarbitone upon delivery as an unventilated control (UVC) group for comparison of lung injury data. UVC lambs were used across multiple studies in our program each year to match flock and environmental characteristics.

Data Acquisition and Analysis

Physiological parameters were recorded at 200 Hz (LabChart V8, AD Instruments, Sydney, Australia), analysed at key time points with EIT data and ΔP, tidal volume (V_T), dynamic respiratory system compliance (C_{dyn}) and minimum (diastole), maximum (systole) and average cerebral blood flow calculated.(1, 10, 13, 16, 17) Dynamic tidal mechanical power (MP_{tidal}) was calculated using the simplified equation of Becher and co-workers: MP_{tidal} (J/kg/min) = 0.098 x V_T x ΔP; V_T was expressed in L/kg.(18) The mechanical energy of the respiratory system (ME_{RS}) was calculated using the equation of Kneyber and co-workers: ME_{RS} (mJ/kg) = 0.098 x (V_T/kg) x ΔP.(19) Ventilator efficacy index (VEI), an index that in the absence of spontaneous breathing integrates ventilation with magnitude of respiratory support, was calculated using the formula: VEI = 3800/(ΔP x f x $PaCO_2$); where 3800 is a CO_2 production constant ($mL.mmHg.kg^{-1}.min^{-1}$), f the ventilator rate and $PaCO_2$ the arterial partial pressure of CO_2.(20-22) Time-course EIT image data were reconstructed

using an anatomically correct custom-built lamb algorithm,(4, 16, 23) filtered to the respiratory domain (IBEX software package, Sentec).(5, 6, 9) This algorithm accounts for the specific shape of the lamb chest, including the greater lung size in the right and dorsal hemithoraces.(4) Change in lung volume (ΔV_L) was calculated from the tidal time-course EIT signal low-pass filtered to the expiratory signal (<0.5 Hz).(3, 15) Global ΔV_L from the pre-aerated state was calibrated from the static pressure-volume curve. Relative ΔV_L (aeration) within the gravity dependent and non-dependent regions was determined from the raw data and weighted to the known pixel (anatomical) contribution of each region to provide a measure of relative aeration states within the lung.(4, 5, 15) The ventral-dorsal centre of ventilation (CoV) was used to define the spatiotemporal distribution of V_T.(16, 24)

Physiological and EIT data reported at key time points were determined from 30 s of consecutive artefact-free data for data from 3 to 15 or 90 min. During the first 3 min of respiratory support, data were determined over 10 s intervals to coincide with each PEEP step in the Dynamic PEEP strategies. Consistent with our previous studies of dynamic PEEP, the values at P_{MAX} and at PEEP 8 cmH_2O on completion of the initial step-wise PEEP decrease were reported as 1 and 2 min rather than the actual time points as these are physiologically more meaningful.(3, 9, 14)

Immediately after completing the protocol, the lungs were removed *en bloc*. Protein concentration of left lung lavage was determined using bicinchroninic protein assay.(25) The right upper lobe was inflation fixed at 20 cmH_2O with 4% paraformaldehyde buffered in PBS solution, underwent a series of dehydration and rehydration washes and was wax embedded prior to preparation of hematoxylin and eosin–stained tissue sections from each of the gravity dependent and non-gravity-dependent zones and assessment using our previously reported standardised criteria.(4, 15, 26, 27) This included first identifying representative lung regions within the centralised field of view (to avoid artefact related to tissue processing) by an investigator unaware of treatment group. 10 standardised

consecutive images were at 10x magnification from a grid pattern and applied to each selected lung region.(23) All image analysis was performed using FIJI software on 10 fields of view.(28)

Proteome Analysis

Access to mass spectrometry facilities over the course of Studies 1 and 2 meant proteome experiments were performed at separate Institutes with moderate variations in protocol.

Sample acquisition: Immediately after animals were killed, the lungs were removed *en bloc*. Lung tissue samples for proteome analysis from the gravity-dependent and non-gravity-dependent zones of the right lower lobe (corresponding to the portion of right lung most included in the field of EIT imaging) were snap frozen and stored at -80 °C until analysis.

Study 1

Protein extraction and digestion: 25 mg of frozen lung tissue was homogenised on ice in lysis buffer (8 M Urea, 50 mM HEPES, 1x Halt protease/phosphatase inhibitor (78440; Thermo Fisher Scientific), pH 8) using the Tissue Tearor™ fitted with a 5 cm probe (BioSpec, OK, USA). Protein extracts were centrifuged at 13,000 rpm for 20 minutes at 4 °C (FA-45-30-11 rotor, Eppendorf 5430 R) and supernatants transferred to low protein-binding tubes. Protein concentration was determined using the Pierce MicroBCA™ protein assay (23235; Thermo Fisher Scientific) according to manufacturer's instructions. The use of this specific assay was due to the reagents used in our lysis buffer, in addition to the dynamic range and volumes used in this protein assay. Protein lysates (25 µg at 0.4 µg/µL concentration) were reduced with 10 mM dithiothritol (DTT) (C-1029-5G, Astral Scientific, Australia) at 450 rpm for 1 h at room temperature (RT, 25 °C) followed by alkylation with 20 mM iodoacetic acid (IAA) (I1149-25G, Sigma-Aldrich, Castle Hill, AU) at 450 rpm for 30 min at RT (light protected). Samples were immediately quenched with 10 mM DTT before being subjected to singlepot, solid-phase-enhanced sample separation (SP3) as previously described.(29, 30)

Magnetic beads were prepared by mixing SpeedBeads™ magnetic carboxylate modified particles (65152105050250, 45152105050250, Cytiva Life Sciences, USA) at 1:1 (v:v) ratio and washing twice with 200 µL MilliQ. Beads were transferred to protein extracts in a 10:1 total bead to protein ratio, with addition of 50% (v/v) ethanol. Samples were mixed at 1000 rpm for 10 min at RT and transferred to a magnetic rack where the supernatant was removed, and protein-bound beads were washed three times with 80% (v/v) ethanol. Samples were reconstituted in 50 mM TEAB pH 8 and digested with MS grade trypsin (enzyme: substrate 1:50, Promega V5113) and Lysyl Endopeptidase (enzyme:substrate 1:100, 125-05061, Wako Pure Chemical Industries) for 18 h, 1000 rpm at 37 °C. Peptide digests were centrifuged at 20,000 g for 1 min, supernatants transferred to fresh low-protein binding tubes, snap frozen and lyophilised via vacuum centrifugation (Savant SPD121P, Thermo Fisher Scientific).

TMT-labelling: Peptides were reconstituted in 100 mM TEAB pH 8.5, quantified using Pierce™ Fluorometric Peptide Assay (#23290, Thermo Fisher Scientific), adjusted to 0.1 µg/µL and TMT-labelled according to manufacturer's instructions with minor modifications. A pooled internal reference channel was prepared across all sample groups. Samples were labelled using tandem mass tag (TMT) TMT11-plex (A34808/06, Lot VL312003, Thermo Fisher Scientific) (9 samples and 2 pooled internal reference channels, combined in equal ratio). TMT tags were prepared in anhydrous acetonitrile (ACN) and transferred to 5 µg of peptide digests in a 1:4 peptide: TMT ratio. Samples were incubated at RT for 2 h at 450 rpm, quenched with 1 µL of 5% hydroxylamine at RT for 15 min at 450 rpm, and combined in equal ratio. Combined, labelled peptides were acidified to 3% formic acid (FA), desalted using solid phase extraction manifold (186002321/186001831, Waters Corporation), eluted in 50% ACN and 0.1% formic acid in MS-grade water, and dried by vacuum centrifugation. Samples were reconstituted in 0.07% trifluoroacetic acid (TFA) in MS-grade water, quantified using Pierce™ Quantitative Peptide Assay (#23275, Thermo Fisher Scientific) and normalized to 0.5 µg/µL.

TMT-based proteomics: TMT-labelled peptides were analysed on a Dionex 3500RS nanoUHPLC coupled to an Q Exactive HF-X benchtop Orbitrap mass spectrometer equipped with nanospray ion source in positive, data-dependent acquisition mode as previously described.(31) Peptides (0.5 µg) were sequentially loaded (Acclaim PepMap100 C18 3 mm beads with 100 Å pore-size, Thermo Fisher Scientific) and separated (1.9-µm particle size C18, 120 Å pore-size, 0.075 × 200 mm, Nikkyo Technos Co. Ltd) and a gradient of 3–80% (0-3 min, ACN containing 0.1% (v/v) FA over 240 min (2-28% over 224 min, 28-80% to 228 min, 80-2% to 234 min) at 300 nl min-1 at 55°C (butterfly portfolio heater, Phoenix S&T). Peptides were injected into the trap column at an isocratic flow of 5 µL/min of 0.1% (v/v) FA for 5 min, applied before switching in-line with the analytical column.

An MS1 scan was acquired from 350–1,500 m/z (120,000 resolution, isolation window of 0.7 Thomsons) in 'top speed' acquisition mode with 3 s cycle time (max IT 250 ms) on the 25 most intense precursor ions; ions with charge states of 2 to 5 were selected. AGC target was set to 3×106 automatic gain control, followed by MS/MS data-dependent acquisition with high-field collision-induced dissociation and detection in the Orbitrap (45,000 resolution, 1×105 AGC, 90-ms injection time, normalised collision energy of 32), with minimum AGC target 4.5×103. Dynamic exclusion was activated for 20 s, with data acquired using Xcalibur v4.5 software.

Study 2

Protein extraction and digestion: 25 mg of lung tissue was homogenised in lysis buffer (same as Study 1) containing 1.4 mm (0.9-2.0 mm) stainless steel bead blend (1:2 ratio; SSB14B, Next Advance, NY, USA) using the Bullet Blender 24 Gold (BB24AU, Next Advance). Samples were homogenised at Speed 10 Time 4, then diluted 1:2 and spun at Speed 2 Time 1. Protein extracts were centrifuged at 13,000 rpm for 20 min at 4 °C and supernatants transferred to fresh low protein-binding tubes.

Protein lysates (25 µg at 0.4 µg/µL concentration) were reduced with 10 mM DTT at 174 rpm (OM11plate shaker, Ratek, VIC, AU) for 1 h at RT, followed by alkylation with 20 mM iodoacetic acid at 174 rpm for 30 min at RT in the dark. Samples were immediately quenched with 10 mM DTT before being subjected to SP3 protocol, as described in Study 1. Following reduction and alkylation, samples were reconstituted in 50 mM TEAB pH 8 and digested with MS grade trypsin protease (1:50 trypsin: protein ratio; 90057, Thermo Fisher Scientific) for 18 h, 387 rpm at 37 °C. Peptide digests were centrifuged at 20,000 g for 1 min, supernatants transferred to fresh low-protein binding tubes, snap frozen and lyophilised via vacuum centrifugation (ISS110 SpeedVac System). Samples were incubated at RT for 2 h at 450 rpm, quenched with 1 µL of 5% hydroxylamine at RT for 15 min at 450 rpm, and combined in equal ratio.

TMT-labelling: Peptides were reconstituted in 100 mM TEAB pH 8.5, quantified using MicroBCA™ assay, adjusted to 1 µg/µL and TMT-labelled. A pooled internal standard for normalisation between runs was prepared by combining 1 µg of peptide from each individual sample. Samples were randomly allocated in TMT11-plex runs (90110/AA37725, Lot XF334595, Thermo Fisher Scientific) (nine samples and two pooled internal standards for each TMT run). TMT tags were prepared in anhydrous acetonitrile (ACN) and transferred to 10 µg of peptide digests in a 1:8 peptide: TMT ratio. Samples were incubated at 155 rpm, RT for 1 h, quenched with 1 µL of 5% hydroxylamine at 155 rpm for 15 min at RT, and combined in equal ratio per TMT11-plex run. Samples were acidified to 3% formic acid (pH<2) prior to sample clean-up using solid phase extraction (SPE) cartridges (186000383, Waters Australia). Following SPE clean-up, samples were lyophilised, reconstituted in 2% ACN/0.05% TFA, centrifuged at 20,000 *g* for 10 min at RT with the top 50% of sample taken for filtration through a 30 kDa centrifugal filter (MRCF0R030, MilliporeSigma, MA, USA). An initial run was performed to normalise the total reporter ion intensity of each multiplexed sample and check labelling efficiency.

LC-MS/MS Analysis: TMT-labelled peptides (450 ng) were analysed by LC-MS/MS at The Mass Spectrometry and Proteomics Facility, Bio21 Institute, Melbourne University. The nano-LC system, Ultimate 3000 RSLC was equipped with an Acclaim Pepmap nano-trap column (C18, 100 Å, 75 m× 2 cm) and an Acclaim Pepmap RSLC analytical column (C18, 100 Å, 75 m × 50 cm) maintained at a temperature of 50 °C. 1 μg of peptide mixture was loaded onto the trap column at an isocratic flow of 5 L/min of 3% CH3CN containing 0.05% TFA for 6 min before the enrichment column was switched in-line with the analytical column. The eluents used for the LC were water with 0.1% (v/v) formic acid and 5% (v/v) dimethyl sulfoxide (DMSO) for solvent A, and ACN with 0.1% (v/v) formic acid and 5% DMSO for solvent B. The gradient used at 300 nL/min was from 3-23% solvent B for 134 min, 23-40% solvent B in 20 min, 40-80% solvent B in 10 min and maintained at 80% for the final 5 min before dropping to 3% solvent B in 1 min and equilibration for 9 min at 3% solvent B prior to the next analysis. MS experiments were performed using a nano electrospray ionisation source at positive mode and Eclipse Orbitrap mass spectrometer. The spray voltages, capillary temperature and S-lens RF level were set to 1.9 kV, 275°C and 30%. MS data was acquired with a 3 s cycle time for one full scan MS spectra and as many data dependent higher-energy collisional dissociation (HCD)-MS/MS spectra as possible. Full scan MS spectra had a m/z of 375-1500, a resolution of 120,000 at m/z 200, an auto gain control (AGC) target value of 4e5 and a maximum ion trapping time of 50 ms. Data dependent HCD-MS/MS of precursor ions (charge states from 2 to 7) was performed using an m/z isolation window of 0.7, first mass at m/z of 110, AGC target value of 1.25e5, normalised collision energy (NCE) of 38%, resolution of 30,000 at m/z 200 and maximum ion trapping time of 54 ms. Raw MS data was acquired using Orbitrap mass analyser. Dynamic exclusion was activated for 30 s.

Studies 1 and 2

Database searching and protein identification: Raw MS data were analysed using MaxQuant (v1.6.14.0/v2.1.3.0, Uniprot Ovis aries (sheep) database; UP000002356, 23111 entries, Feb/Aug 2023) using the built-in search engine Andromeda(32) with a contaminants database employed. Cysteine carbamidomethylation was selected as a fixed modification and N-terminal acetylation and methionine oxidations as variable modifications. Data was processed using LysC (Study 1 only) and/or trypsin/P as the proteolytic enzymes with up to 2 missed cleavage sites allowed. For TMT-based analyses, reporter ion MS2 (11plex TMT) settings were employed. False discovery rate (FDR) for peptide and protein spectrum match was set at 0.01. Peptides were identified with an initial precursor mass deviation of up to 7 ppm and a fragment mass deviation of 20 ppm. Quantification was performed based on intensity of the reporter ion, with a mass tolerance of 0.003 Da and correction for isotopic impurities. 'Match between runs' algorithm was performed.

TMT normalisation: Search results and TMT reporter ion intensities were exported as text files. Within the data set, unreliable protein groups (razor peptides <1, contaminants, peptides only identified by site, reversed) and proteins with reporter ion intensities missing from all samples in a 11-plex run were removed. A 2-step normalisation procedure was employed as per Plubell et al., in RStudio (v4.1.2).(33) The first normalisation was applied within each 11-plex experiment. The grand total reporter ion intensity for each channel was multiplied by global scaling factors to adjust its total intensity to the average total intensity across the 11 channels. This corrects for small sample loading and labelling reaction efficiency differences. Secondly, common, pooled internal standards were used to normalise reporter ion intensities of proteins between different TMT experiments. This allowed preservation of individual intensity-scale measurements and avoided calculation of relative intensity measures such as ratios or percentages within each TMT experiment. To accomplish this, scaling factors which were calculated from the internal standards in each run (minimum 2 standards/run) and used to adjust the summed reporter ion intensities for each protein in the remaining eight experimental samples in each TMT experiment.(31, 34)

Statistical Analysis

8 to 10 lambs/group were able to demonstrate differences in our histological markers following 15 min of PPV,(13, 35) and 15-20/gp following 90 min PPV at each allocated gestation (both 80% type 1 error, p=0.05).(1) All non-proteomics data were first tested for normality prior to analysis and investigated with either one-way ANOVA, Kruskal-Wallis test or mixed effects analysis (ventilation strategy and time as variables) and appropriate post-test analysis. Statistical analysis was performed with PRISM 9 (GraphPad Software, San Diego, CA). Proteome analysis to identify differentially expressed proteins (DEPs; identified as having a significant difference in protein abundance in ventilated groups compared with the unventilated control [UVC] group) was performed in EdgeR software package in RStudio.(36, 37) Significance was confirmed by QL F-testing with multiplicity correction applied using Benjamin-Hochberg method on the p-values to control the FDR, with significance set FDR<5%.

Proteome Bioinformatic Analysis

Multivariate analysis using principal component analysis (PCA) was performed in ClustVis.(38) Gene set enrichment analysis (GSEA) to identify enriched Reactome pathways associated with the proteome datasets were identified using WebGestalt, with weight-set coverage redundancy applied (filtered for pathways containing ≥5 proteins) and FDR<0.05 considered significant.(39) PANTHER software (v18.0) was used to characterise biological and cellular processes, and protein classes of DEPs within the proteome dataset.(40) Protein-protein network interaction generated in STRING (v12).(41) Similar to previous studies, (35, 42) to address ovine database limitations in analysis tools, homology of sheep to human proteins was assessed by NCBI Basic Local Alignment Search Tool (BLAST); with 93% of proteins exhibiting homology ≥75%. The mass spectrometry proteomics data have been deposited to the ProteomeXchange Consortium

(http://proteomecentral.proteomexchange.org) via the PRIDE partner repository (43) with the dataset identifier PXD041917 (Study 1) and PXD050305 (Study 2).

REFERENCES

1. Tingay DG, Pereira-Fantini PM, Oakley R, McCall KE, Perkins EJ, Miedema M, et al. Gradual Aeration at Birth Is More Lung Protective Than a Sustained Inflation in Preterm Lambs. *Am J Respir Crit Care Med.* 2019;200(5):608-16.
2. Tingay DG, Togo A, Pereira-Fantini PM, Miedema M, McCall KE, Perkins EJ, et al. Aeration strategy at birth influences the physiological response to surfactant in preterm lambs. *Arch Dis Child Fetal Neonatal Ed.* 2019;104(6):F587-F93.
3. Tingay DG, Rajapaksa A, Zannin E, Pereira-Fantini PM, Dellaca RL, Perkins EJ, et al. Effectiveness of individualized lung recruitment strategies at birth: an experimental study in preterm lambs. *Am J Physiol Lung Cell Mol Physiol.* 2017;312(1):L32-L41.
4. Tingay DG, Rajapaksa A, Zonneveld CE, Black D, Perkins EJ, Adler A, et al. Spatiotemporal Aeration and Lung Injury Patterns Are Influenced by the First Inflation Strategy at Birth. *Am J Respir Cell Mol Biol.* 2016;54(2):263-72.
5. McCall KE, Waldmann AD, Pereira-Fantini P, Oakley R, Miedema M, Perkins EJ, et al. Time to lung aeration during a sustained inflation at birth is influenced by gestation in lambs. *Pediatr Res.* 2017;82(4):712-20.
6. Miedema M, McCall KE, Perkins EJ, Sourial M, Bohm SH, Waldmann A, et al. First Real-Time Visualization of a Spontaneous Pneumothorax Developing in a Preterm Lamb Using Electrical Impedance Tomography. *Am J Respir Crit Care Med.* 2016;194(1):116-8.
7. Sweet DG, Carnielli VP, Greisen G, Hallman M, Klebermass-Schrehof K, Ozek E, et al. European Consensus Guidelines on the Management of Respiratory Distress Syndrome: 2022 Update. *Neonatology.* 2023;120(1):3-23.
8. Madar J, Roehr CC, Ainsworth S, Ersdal H, Morley C, Rudiger M, et al. European Resuscitation Council Guidelines 2021: Newborn resuscitation and support of transition of infants at birth. *Resuscitation.* 2021;161:291-326.

9. Miedema M, Waldmann A, McCall KE, Bohm SH, van Kaam AH, and Tingay DG. Individualized Multiplanar Electrical Impedance Tomography in Infants to Optimize Lung Monitoring. *Am J Respir Crit Care Med.* 2017;195(4):536-8.

10. Tingay DG, Bhatia R, Schmolzer GM, Wallace MJ, Zahra VA, and Davis PG. Effect of sustained inflation vs. stepwise PEEP strategy at birth on gas exchange and lung mechanics in preterm lambs. *Pediatr Res.* 2014;75(2):288-94.

11. Hillman NH, Kemp MW, Noble PB, Kallapur SG, and Jobe AH. Sustained inflation at birth did not protect preterm fetal sheep from lung injury. *Am J Physiol Lung Cell Mol Physiol.* 2013;305(6):L446-53.

12. Hillman NH, Nitsos I, Berry C, Pillow JJ, Kallapur SG, and Jobe AH. Positive end-expiratory pressure and surfactant decrease lung injury during initiation of ventilation in fetal sheep. *Am J Physiol Lung Cell Mol Physiol.* 2011;301(5):L712-20.

13. Tingay DG, Fatmous M, Kenna K, Dowse G, Douglas E, Sett A, et al. Inflating Pressure and Not Expiratory Pressure Initiates Lung Injury at Birth in Preterm Lambs. *Am J Respir Crit Care Med.* 2023;208(5):589-99.

14. Tingay DG, Polglase GR, Bhatia R, Berry CA, Kopotic RJ, Kopotic CP, et al. Pressure-limited sustained inflation vs. gradual tidal inflations for resuscitation in preterm lambs. *J Appl Physiol.* 2015;118(7):890-7.

15. Tingay DG, Lavizzari A, Zonneveld CE, Rajapaksa A, Zannin E, Perkins E, et al. An individualized approach to sustained inflation duration at birth improves outcomes in newborn preterm lambs. *Am J Physiol Lung Cell Mol Physiol.* 2015;309(10):L1138-49.

16. Frerichs I, Amato MB, van Kaam AH, Tingay DG, Zhao Z, Grychtol B, et al. Chest electrical impedance tomography examination, data analysis, terminology, clinical use and recommendations: consensus statement of the TRanslational EIT developmeNt stuDy group. *Thorax.* 2017;72(1):83-93.

17. Dahm SI, Kenna KR, Stewart D, Pereira-Fantini PM, McCall KE, Perkins EJ, et al. Aeration strategy at birth does not impact carotid haemodynamics in preterm lambs. *Pediatr Res.* 2023;93(5):1226-32.

18. Becher T, van der Staay M, Schadler D, Frerichs I, and Weiler N. Calculation of mechanical power for pressure-controlled ventilation. *Intensive Care Med.* 2019;45(9):1321-3.

19. Kneyber MCJ, Ilia S, Koopman AA, van Schelven P, van Dijk J, Burgerhof JGM, et al. Energy transmission in mechanically ventilated children: a translational study. *Crit Care.* 2020;24(1):601.

20. Notter RH, Egan EA, Kwong MS, Holm BA, and Shapiro DL. Lung surfactant replacement in premature lambs with extracted lipids from bovine lung lavage: effects of dose, dispersion technique, and gestational age. *Pediatr Res.* 1985;19(6):569-77.

21. Bach KP, Kuschel CA, Oliver MH, and Bloomfield FH. Ventilator gas flow rates affect inspiratory time and ventilator efficiency index in term lambs. *Neonatology.* 2009;96(4):259-64.

22. Ikegami M, Jobe AH, Newnham J, Polk DH, Willet KE, and Sly P. Repetitive prenatal glucocorticoids improve lung function and decrease growth in preterm lambs. *Am J Respir Crit Care Med.* 1997;156(1):178-84.

23. Adler A, Arnold JH, Bayford R, Borsic A, Brown B, Dixon P, et al. GREIT: a unified approach to 2D linear EIT reconstruction of lung images. *Physiol Meas.* 2009;30(6):S35-55.

24. Frerichs I, Dargaville PA, van Genderingen H, Morel DR, and Rimensberger PC. Lung volume recruitment after surfactant administration modifies spatial distribution of ventilation. *Am J Respir Crit Care Med.* 2006;174(7):772-9.

25. Lowry OH, Rosebrough NJ, Farr AL, and Randall RJ. Protein measurement with the Folin phenol reagent. *J Biol Chem.* 1951;193(1):265-75.

26. Schneider CA, Rasband WS, and Eliceiri KW. NIH Image to ImageJ: 25 years of image analysis. *Nat Meth.* 2012;9(7):671-5.

27. Oakley RB, Tingay DG, McCall KE, Perkins EJ, Sourial M, Dargaville PA, et al. Gestational Age Influences the Early Microarchitectural Changes in Response to Mechanical Ventilation in the Preterm Lamb Lung. *Front Pediatr.* 2019;7:325.

28. Schindelin J, Arganda-Carreras I, Frise E, Kaynig V, Longair M, Pietzsch T, et al. Fiji: an open-source platform for biological-image analysis. *Nat Methods.* 2012;9(7):676-82.

29. Fatmous M, Rai A, Poh QH, Salamonsen LA, and Greening DW. Endometrial small extracellular vesicles regulate human trophectodermal cell invasion by reprogramming the phosphoproteome landscape. *Front Cell Dev Biol.* 2022;10:1078096.

30. Claridge B, Rai A, Fang H, Matsumoto A, Luo J, McMullen JR, et al. Proteome characterisation of extracellular vesicles isolated from heart. *Proteomics.* 2021;21(13-14):e2100026.

31. Greening DW, Notaras M, Chen M, Xu R, Smith JD, Cheng L, et al. Chronic methamphetamine interacts with BDNF Val66Met to remodel psychosis pathways in the mesocorticolimbic proteome. *Mol Psychiatry.* 2021;26(8):4431-47.

32. Cox J, and Mann M. MaxQuant enables high peptide identification rates, individualized p.p.b.-range mass accuracies and proteome-wide protein quantification. *Nat Biotechnol.* 2008;26(12):1367-72.

33. Plubell DL, Wilmarth PA, Zhao Y, Fenton AM, Minnier J, Reddy AP, et al. Extended Multiplexing of Tandem Mass Tags (TMT) Labeling Reveals Age and High Fat Diet Specific Proteome Changes in Mouse Epididymal Adipose Tissue. *Mol Cell Proteomics.* 2017;16(5):873-90.

34. Notaras M, Lodhi A, Dundar F, Collier P, Sayles NM, Tilgner H, et al. Schizophrenia is defined by cell-specific neuropathology and multiple neurodevelopmental mechanisms in patient-derived cerebral organoids. *Mol Psychiatry.* 2022;27(3):1416-34.

35. Pereira-Fantini PM, Pang B, Byars SG, Oakley RB, Perkins EJ, Dargaville PA, et al. Preterm Lung Exhibits Distinct Spatiotemporal Proteome Expression at Initiation of Lung Injury. *Am J Respir Cell Mol Biol.* 2019;61(5):631-42.

36. Lun AT, Chen Y, and Smyth GK. It's DE-licious: A Recipe for Differential Expression Analyses of RNA-seq Experiments Using Quasi-Likelihood Methods in edgeR. *Methods Mol Biol.* 2016;1418:391-416.

37. Robinson MD, McCarthy DJ, and Smyth GK. edgeR: a Bioconductor package for differential expression analysis of digital gene expression data. *Bioinformatics.* 2010;26(1):139-40.

38. Metsalu T, and Vilo J. ClustVis: a web tool for visualizing clustering of multivariate data using Principal Component Analysis and heatmap. *Nucleic Acids Res.* 2015;43(W1):W566-70.

39. Liao Y, Wang J, Jaehnig EJ, Shi Z, and Zhang B. WebGestalt 2019: gene set analysis toolkit with revamped UIs and APIs. *Nucleic Acids Res.* 2019;47(W1):W199-W205.

40. Thomas PD, Ebert D, Muruganujan A, Mushayahama T, Albou LP, and Mi H. PANTHER: Making genome-scale phylogenetics accessible to all. *Protein Sci.* 2022;31(1):8-22.

41. Szklarczyk D, Gable AL, Nastou KC, Lyon D, Kirsch R, Pyysalo S, et al. The STRING database in 2021: customizable protein-protein networks, and functional characterization of user-uploaded gene/measurement sets. *Nucleic Acids Res.* 2021;49(D1):D605-D12.

42. Pereira-Fantini PM, Byars SG, McCall KE, Perkins EJ, Oakley RB, Dellaca RL, et al. Plasma proteomics reveals gestational age-specific responses to mechanical ventilation and identifies the mechanistic pathways that initiate preterm lung injury. *Sci Rep.* 2018;8(1):12616.

43. Perez-Riverol Y, Bai J, Bandla C, Hewapathirana S, García-Seisdedos D, Kamatchinathan S, Kundu D, Prakash A, Frericks-Zipper A, Eisenacher M, Walzer M, Wang S, Brazma A, Vizcaíno JA (2022). The PRIDE database resources in 2022: A Hub for mass spectrometry-based proteomics evidences. *Nucleic Acids Res* 50(D1):D543-D552

SUPPLEMENTARY RESULTS

Online Supplementary Table E1. Characteristics of included subjects*

	N	Fetal Characteristics									Post-mortem respiratory characteristics	
		Gestation (days)	Weight (g)	Sex (F:M)	Parity (S:T)	Fetal Fluid (mL/kg)	Fetal arterial blood gas				Static C_{RS} (mL/kg/cmH_2O)	Lung weight/body weight (g/kg)
							pH	PaO_2 (mmHg)	$PaCO_2$ (mmHg)	BE		
**15 min PPV + 30 min Apnoea (placental support)**												
**4 L/min**	9	125 (123,126)	2873 (297)	3:5	4:4	8.9 (6.8)	7.39 (0.05)	23.0 (3.5)	43.1 (3.5)†	1.1 (3.3)	0.70 (0.18)	30.8 (8.2)
**6 L/min**	10	124.5 (123, 126)	3099 (484)	3:7	4:6	7.4 (6.1)	7.37 (0.06)	22.1 (4.3)	46.3 (6.2)	1.2 (3.5)	0.60 (0.18)	29.3 (5.9)
**8-10 L/min**	8	124 (123, 126)	3166 (206)	0:8	4:5	12.3 (4.5)	7.38 (0.03)	24.5 (3.7)	44.1 (3.9)	0.3 (1.8)	0.58 (0.15)	32.1 (3.2)
**UVC**	13	126 (125, 127)‡	3271 (415)	5:8	4:9	N/A	7.35 (0.04)	25.0 (9.4)	50.5 (7.1)	1.4 (2.1)	N/A	30.0 (4.3)
**90 min PPV**												
**4-6 L/min**	15	128 (126, 129)	3315 (458)	8:7	1:14	12.0 (7.4)	7.35 (0.06)	22.4 (3.9)	46.6 (4.5)	-0.2 (3.0)	1.33 (0.28)	31.0 (4.4)
**8-10 L/min**	15	127 (126, 129)	3189 (490)	10:5	4:11	49.7 (22.7)	7.34 (0.05)	30.3 (27.1)	46.5 (4.0)	-0.9 (2.7)	1.19 (0.15)	32.5 (3.4)
**UVC**	11	127 (127, 130)	3183 (373)	7:4	3:8	N/A	7.35 (0.05)	35.3 (12.5)	46.9 (5.2)	0.0 (3.8)	N/A	32.3 (5.4)

F; female, M; male, S; singleton, T; twin/triplet, C_{RS}; static respiratory system compliance, PaO_2; partial pressure of arterial oxygen, $PaCO_2$; partial pressure of arterial carbon dioxide; BE, base excess. All data mean (SD) or ratio except gestation; median (range). †p=0.03 4 L/min vs UVC (one-way ANOVA), ‡p=0.001 against all other groups (Kruskal-Wallis test).***Exclusions. Study 1**: 3 total (1; $F4_{15}$, 1; $F6_{15}$, 1; $F8_{15}$), **Study 2**: 4 total (2; $F8_{90}$, 1; $F4_{90}$ and 1; UVC). Reasons for exclusion were fetal acidosis (pH <7.20; n=2), persistent metabolic acidosis during placental support PPV (n=2) or air leak (2; $F8_{90}$, 1; $F4_{90}$).

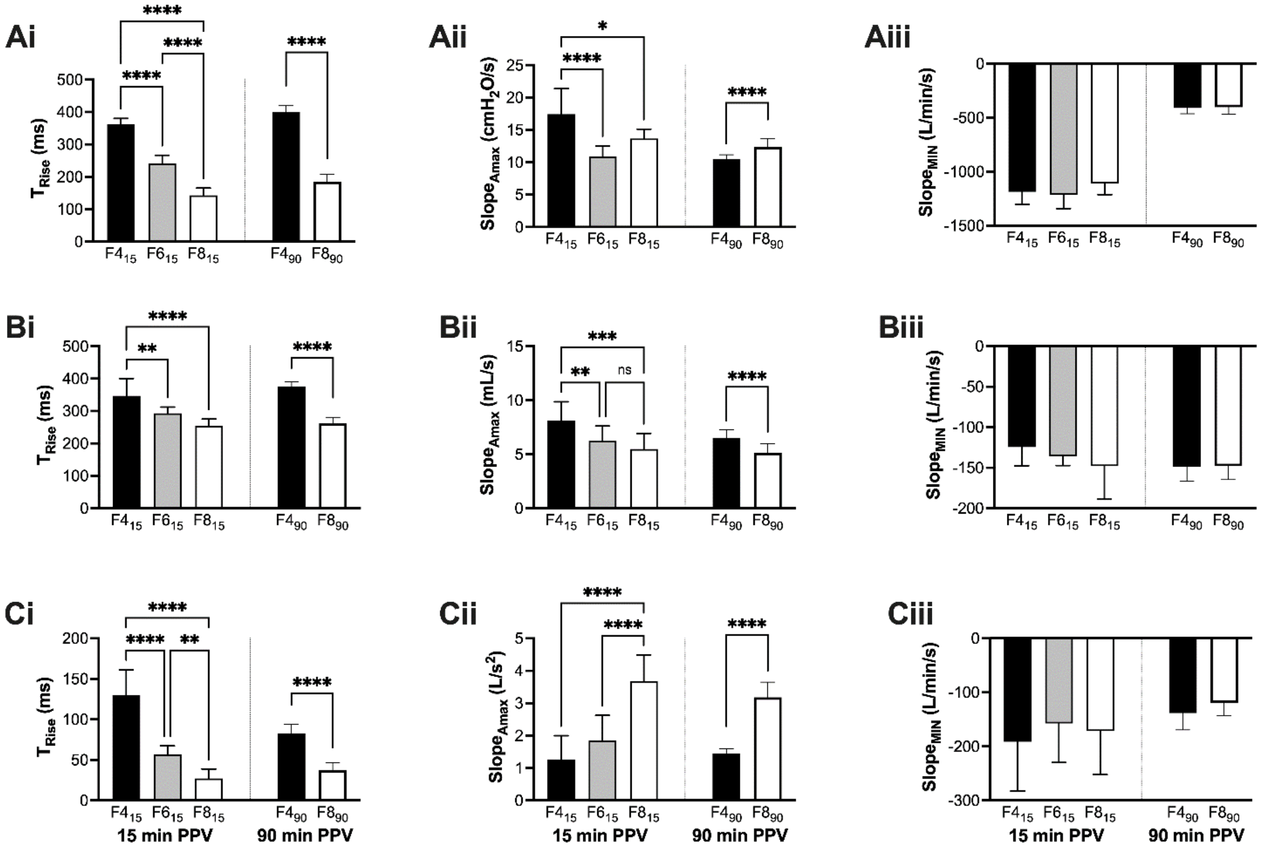

Online Supplementary Figure E1. Additional characteristics of tidal lung motion. Pressure (**Ai-iii**), tidal volume (**Bi-iii**) and flow (**Ci-iii**) wave time at maximum inspiratory slope (T_{Rise}; **Ai-Ci**), average maximum inspiratory slope ($Slope_{Amax}$) for pressure (**Aii**) and tidal volume (**Bii**) and gas flow (**Cii**; represents the average inspiratory acceleration of volume into the lung) and maximum speed of pressure (Aiii), tidal volume (**Biii**) and gas flow (**Ciii**; represents the average inspiratory acceleration of volume into the lung) during expiration ($Slope_{MIN}$). Black bars represent 4 L/min ($F4_{15}$ and $F4_{90}$) groups, grey bars 6 L/min ($F6_{15}$) and white 8-10 L/min ($F8_{15}$ and $F8_{90}$) bias flow strategy for each ventilation period. Bars mean and error bars SD. * $p<0.05$, ** $p<0.01$, *** $p<0.001$, **** $p<0.0001$ Tukey post-test (one-way ANOVA) or t-test.

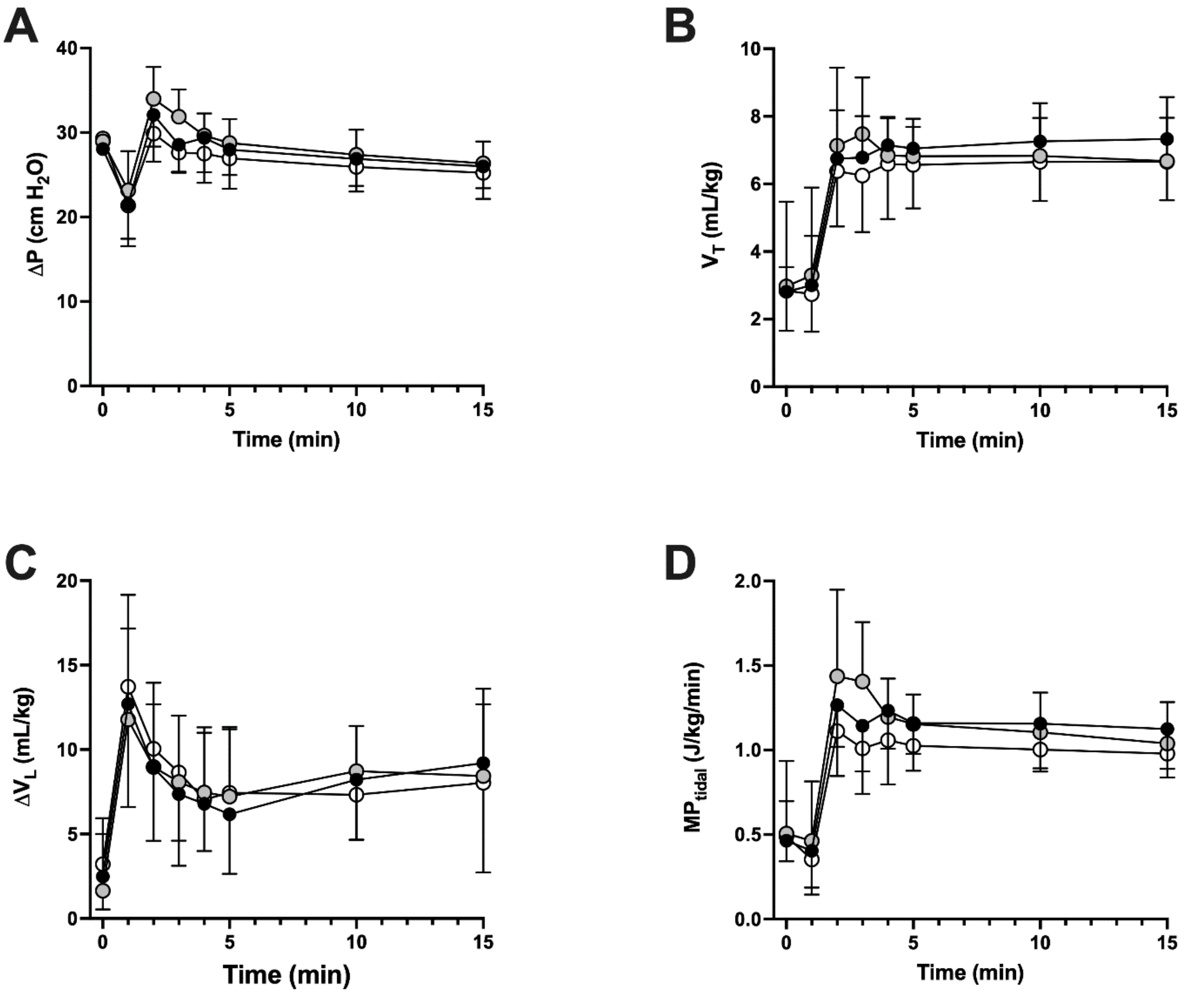

Online Supplementary Figure E2. Additional lung mechanics and volume data for the 15 min ventilation period study. Driving pressure (ΔP (PIP-PEEP); **A**), V_T (**B**), change in global lung volume (ΔV_L; **C**) and tidal mechanical power (MP_{tidal}; **D**). There was no difference between the 4, 6 and 8-10 L/min groups for all parameters and time points (mixed effect model with Tukey post-tests). Black circles represent 4 L/min ($F4_{15}$), grey circles 6 L/min ($F6_{15}$) and white circles 8-10 L/min ($F8_{15}$) bias flow strategy. Data mean and SD.

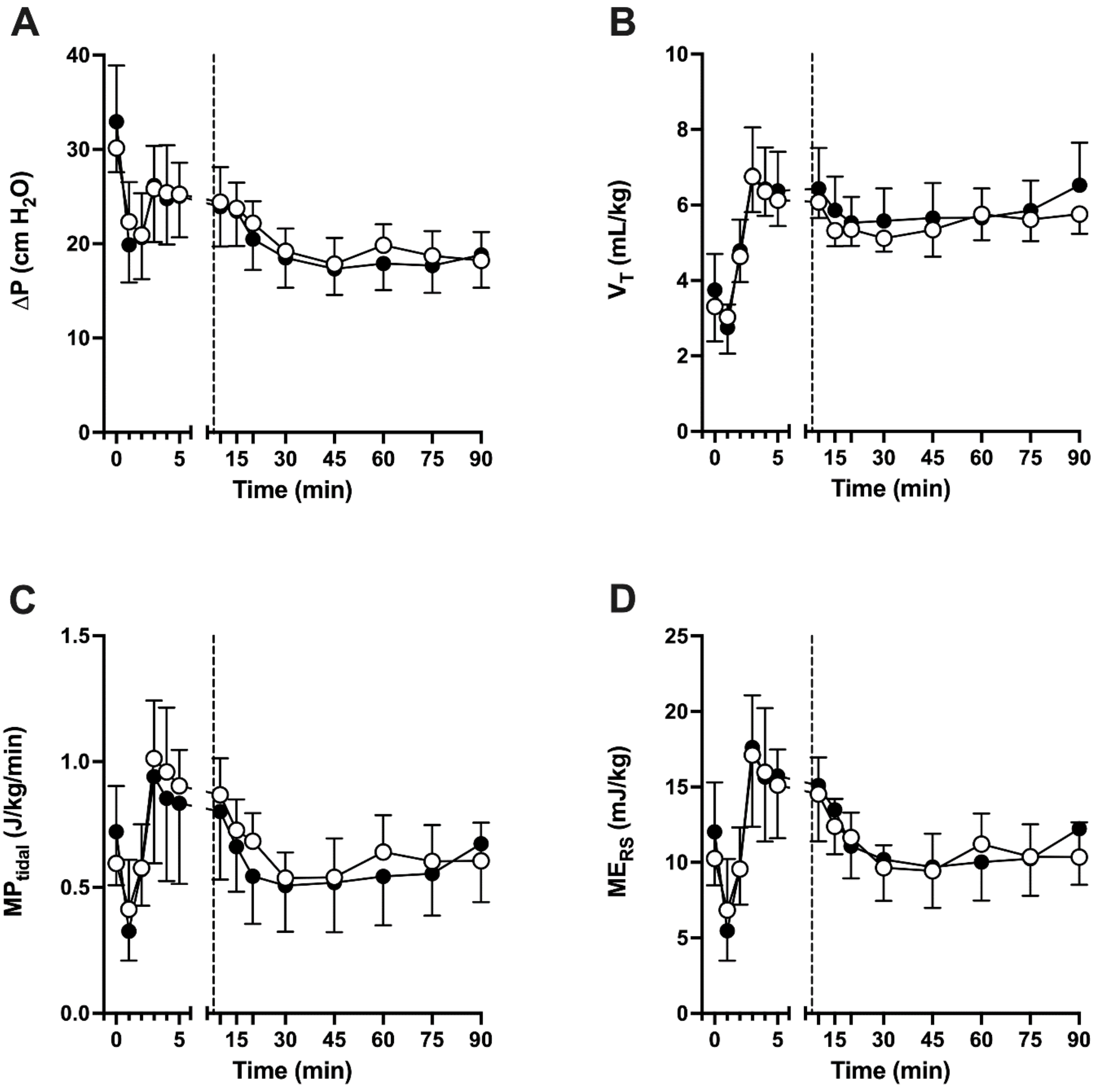

Online Supplementary Figure E3. Additional lung mechanics and volume data for the 90 min ventilation period study. Driving pressure (ΔP (PIP-PEEP); **A**), V_T (**B**), tidal mechanical power (MP_{tidal}; **C**) and mechanical energy of the respiratory system (ME_{RS}; **D**). There was no difference between the 4-6 and 8-10 L/min groups for all parameters and time points (mixed effect model with Tukey post-tests). Black circles represent 4 L/min ($F4_{90}$) and white circles 8-10 L/min ($F8_{90}$) bias flow strategy. Data mean and SD.

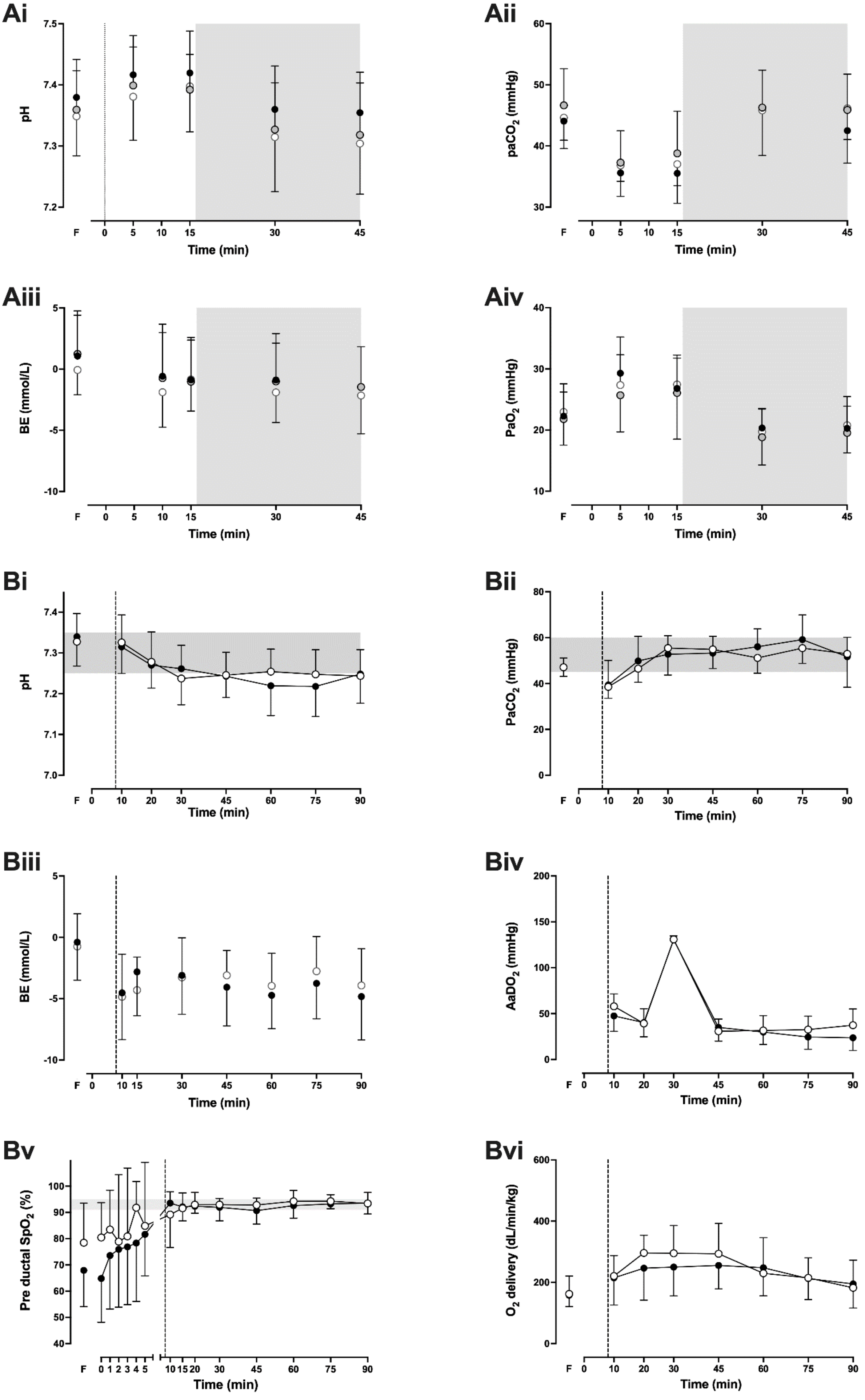
Ai
pH
Time (min)
Aii
paCO2 (mmHg)
Time (min)
Aiii
BE (mmol/L)
Time (min)
Aiv
PaO2 (mmHg)
Time (min)
Bi
pH
Time (min)
Bii
PaCO2 (mmHg)
Time (min)
Biii
BE (mmol/L)
Time (min)
Biv
AaDO2 (mmHg)
Time (min)
Bv
Pre ductal SpO2 (%)
Time (min)
Bvi
O2 delivery (dL/min/kg)
Time (min)

Online Supplementary Figure E4. Arterial blood gas and oxygenation data for 15 min (with placental support; A) and 90 min (off placental support from 3 min; B) ventilation periods. pH (**i**), arterial partial pressure of carbon dioxide ($PaCO_2$; **ii**), base excess (BE; **iii**), partial arterial pressure of oxygen (PaO_2; **Aiv**) or alveolar-oxygen gradient ($AaDO_2$; **Biv**) peripheral oxygen saturation (SpO_2; **v**) and carotid artery oxygen delivery (**vi**). SpO_2 and carotid artery oxygen delivery data for 90 min ventilation period only. Vertical grey shading in 15 min ventilation (A series) panels represent time apnoeic time period on placental support. Horizontal grey shading represents target range for parameter. There was no difference between all flow groups within each ventilation period for all parameters and time points (mixed effect model with Tukey post-tests). Black symbols represent 4 L/min ($F4_{15}$ and $F4_{90}$) groups, grey symbols 6 L/min ($F6_{15}$) and white symbols 8-10 L/min ($F8_{15}$ and $F8_{90}$) bias flow strategy for each ventilation period. Data mean and SD.

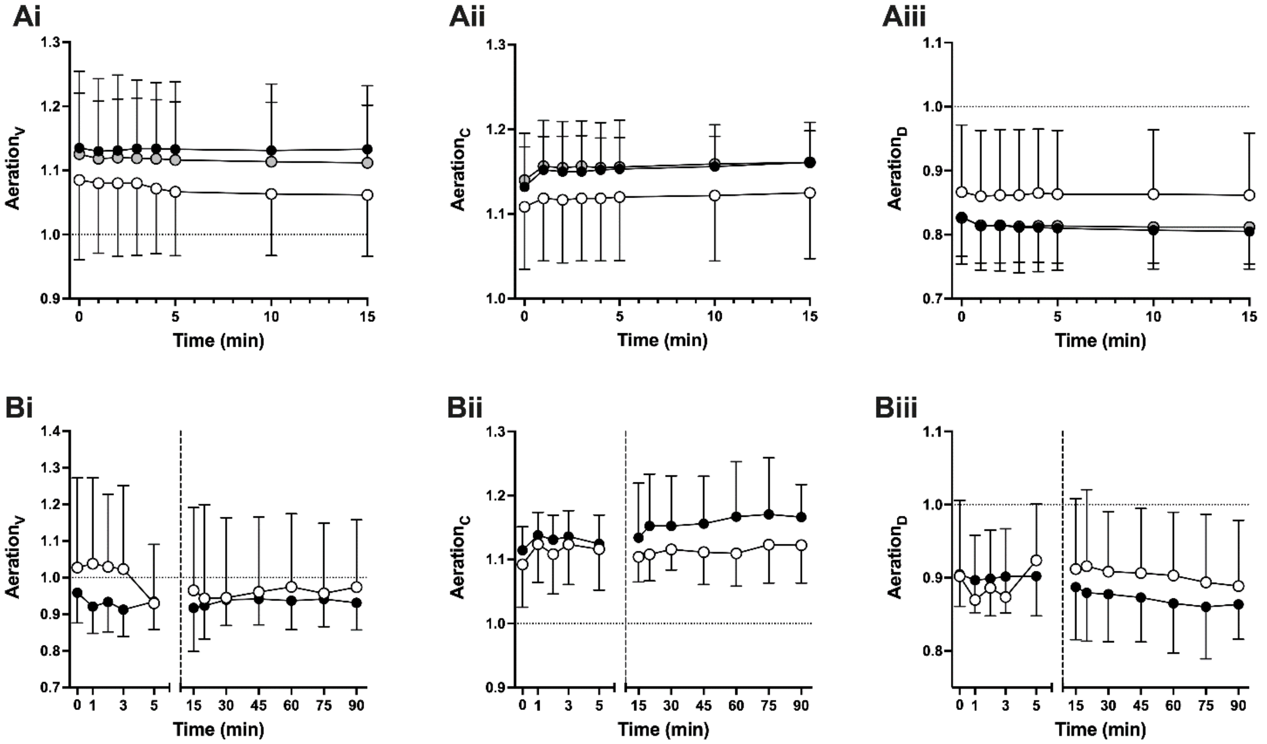

Online Supplementary Figure E5. Additional regional aeration data. Relative lung aeration in the most ventral (**i**), central (**ii**) and most dorsal (**iii**) thirds of the lungs along the gravity-dependent plane for the 15 min (**A**) and 90 min (**B**) ventilation periods. Relative aeration expressed as the ratio of measured aeration to ideal aeration distribution within the lung. A value of 1.0 (ideal) indicates aeration is distributed evenly within the lungs. A value >1.0 indicates relatively greater aeration in the lung region. There was no statistical difference between all flow groups within each ventilation period for all parameters and time points (mixed effect model with Tukey post-tests), although the 8-10 L/min had a trend towards more uniform gravity-dependent aeration. Black symbols represent 4 L/min ($F4_{15}$ and $F4_{90}$) groups, grey symbols 6 L/min ($F6_{15}$) and white symbols 8-10 L/min ($F8_{15}$ and $F8_{90}$) bias flow strategy for each ventilation period. Data mean and SD.

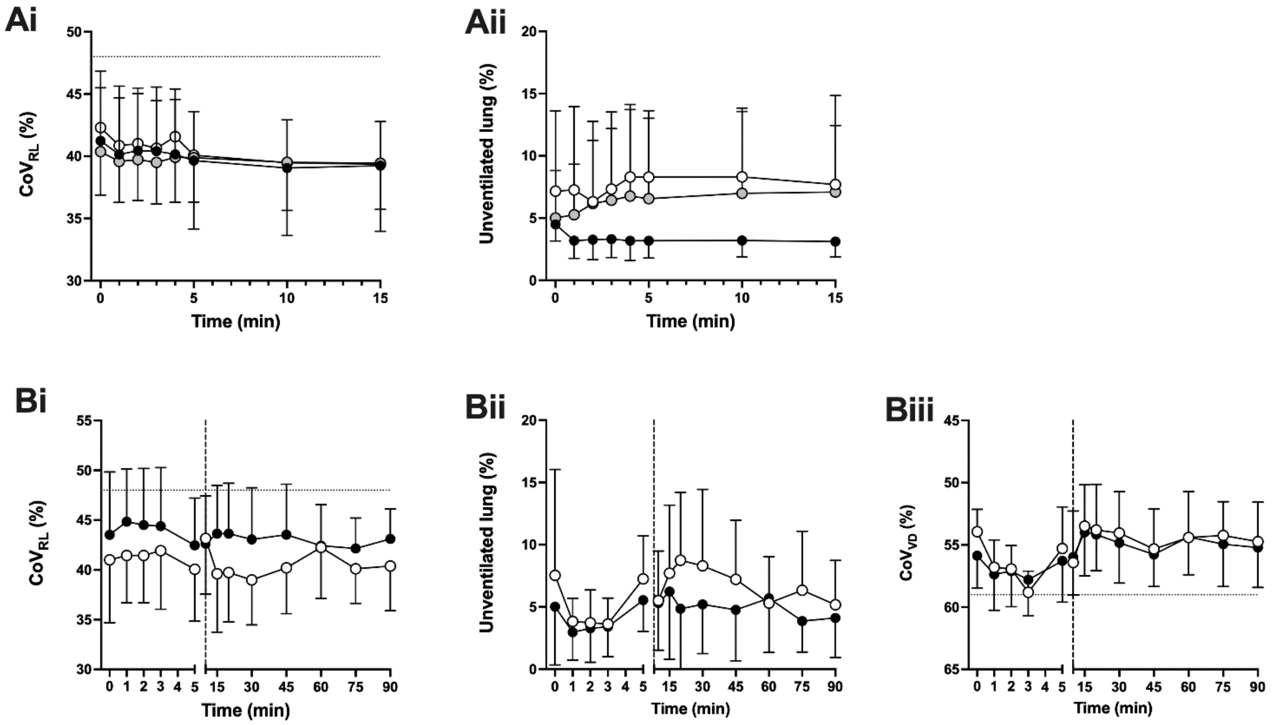

Online Supplementary Figure E6. Additional regional ventilation data. Right to left lung centre of ventilation (CoV_{RL}) for the 15 min (**Ai**) and 90 min (**Bi**) ventilation periods, percentage of lung regions with no apparent tidal ventilation (also called silent spaces; **Aii** and **Bii**) and ventral to dorsal (gravity-dependent) centre of ventilation (CoV_{VD}) for the 90 min ventilation period only (**Biii**). Dotted lines in each figure indicate the CoV during uniform ventilation. Values lower than the value of uniform CoV indicate greater ventilation in the right or ventral (non-dependent) lung compared to the left or dorsal (dependent) lung accordingly. There was no difference between all flow groups within each ventilation period for all parameters and time points (mixed effect model with Tukey post-tests), although the percentage of unventilated lung regions trended to be less with the 4 and 4-6 L/min groups. Black symbols represent 4 L/min ($F4_{15}$ and $F4_{90}$) groups, grey symbols 6 L/min ($F6_{15}$) and white symbols 8-10 L/min ($F8_{15}$ and $F8_{90}$) bias flow strategy for each ventilation period. Data mean and SD.

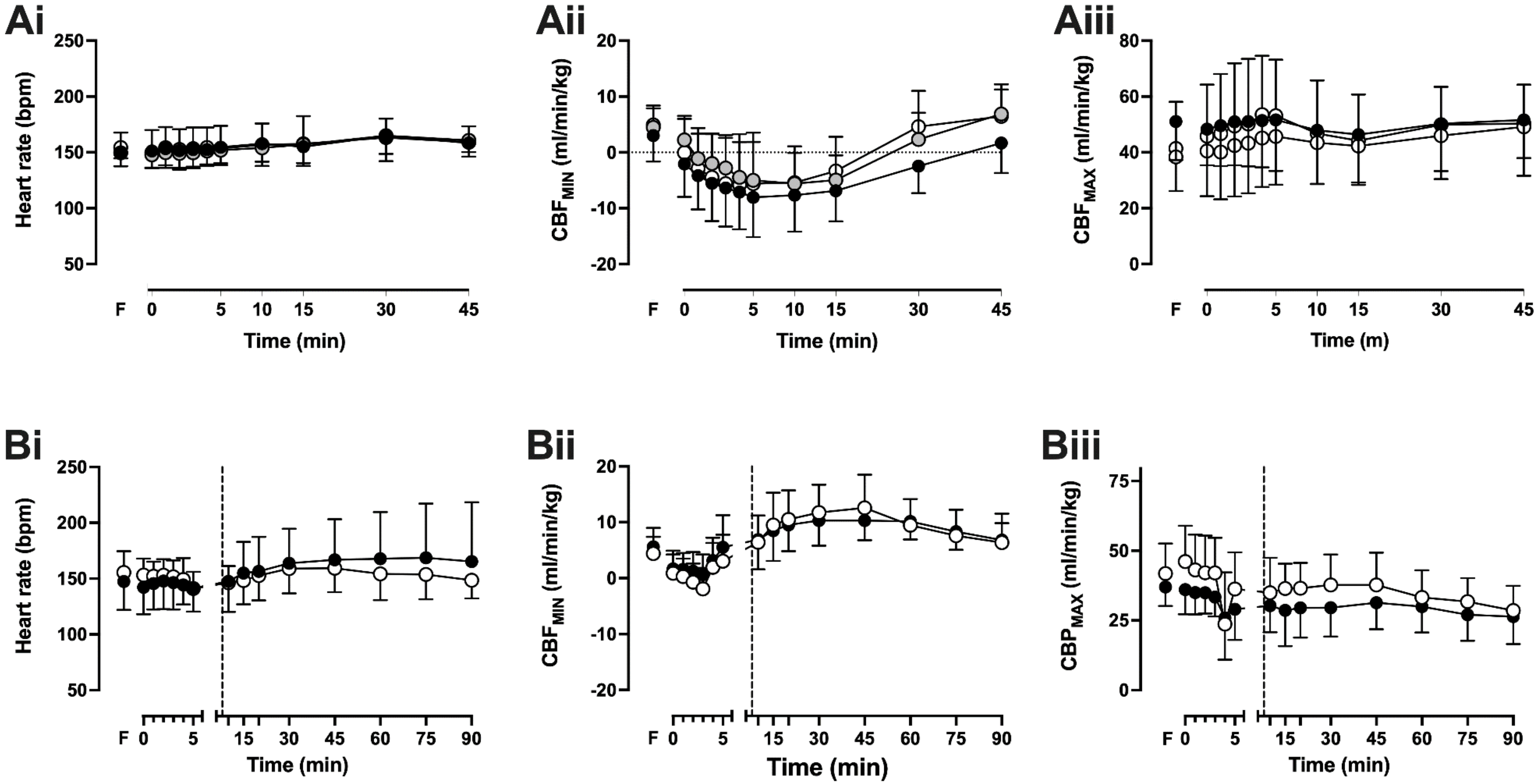

Online Supplementary Figure E7. Cardiovascular Data for lambs ventilated for 15 min (A; with placental support) and 90 min (B; off placental support from 3 min) ventilation periods. Heart rate (**i**), minimum (**ii**) and maximum (**iii**) cerebral blood flow (CBF; referenced to body weight). F represents fetal recording before lung aeration. Black symbols represent 4 L/min ($F4_{15}$ and $F4_{90}$) groups, grey symbols 6 L/min ($F6_{15}$) and white symbols 8-10 L/min ($F8_{15}$ and $F8_{90}$) bias flow strategy for each ventilation period. Data mean and SD.

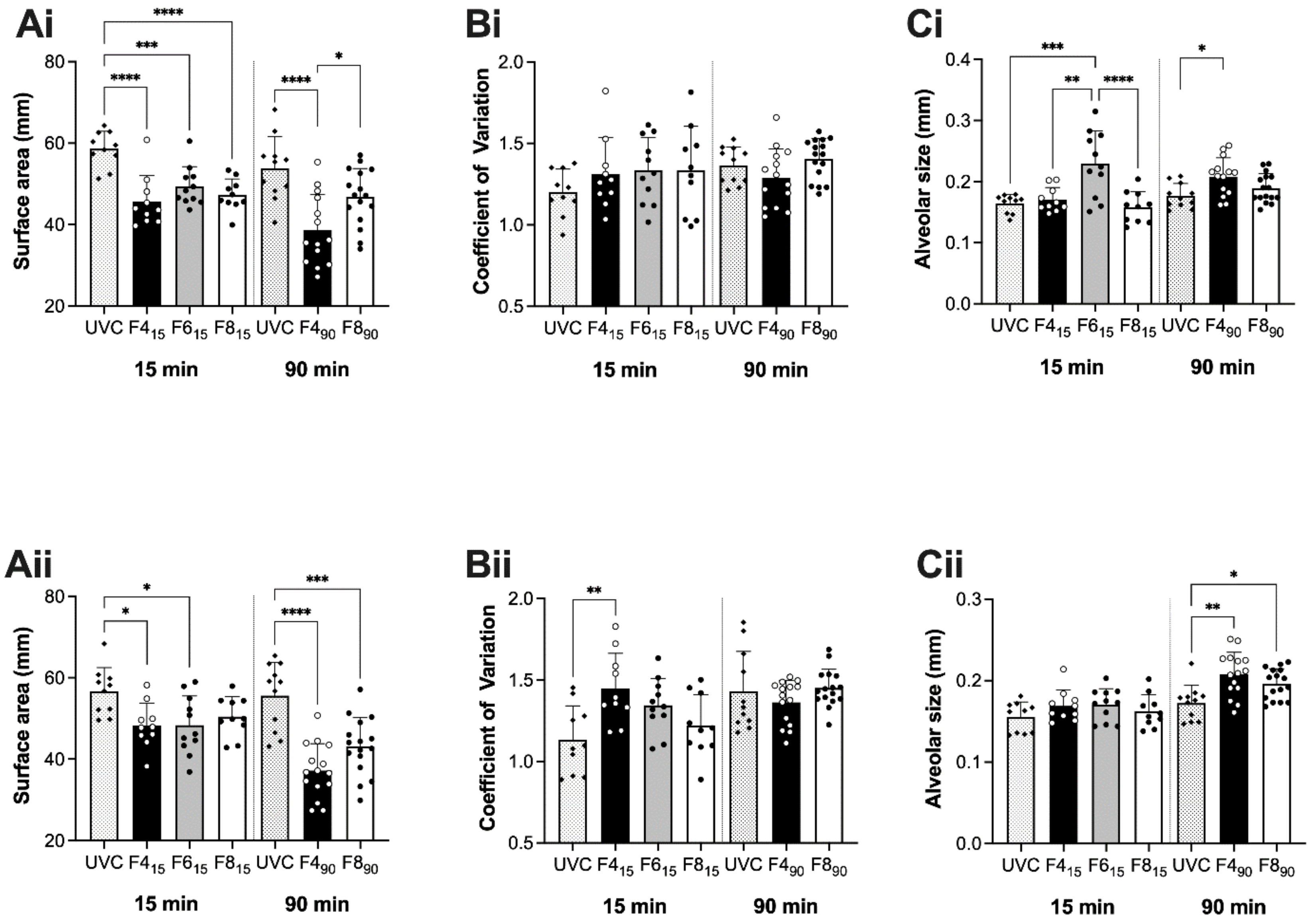
Ai
Surface area (mm)
UVC F4$_{15}$ F6$_{15}$ F8$_{15}$ UVC F4$_{90}$ F8$_{90}$
15 min
90 min
Bi
Coefficient of Variation
Ci
Alveolar size (mm)
Aii
Bii
Cii

Online Supplementary Figure E8. Additional histology data for lambs ventilated for 15 min (with placental support) and 90 min (off placental support from 3 min) ventilation periods in the non-gravity dependent (Ai-Di) and gravity dependent (Aii-Dii) lung. A) number of alveoli per field of view (FOV; lower value more aerated alveoli), **B)** coefficient of variation of the number of alveolar per FOV and **C)** alveolar size (circumference). All 10x magnification. Dotted bars represent unventilated fetal control (UVC) lambs, black bars 4 L/min ($F4_{15}$ and $F4_{90}$) groups, grey bars 6 L/min ($F6_{15}$) and white 8-10 L/min ($F8_{15}$ and $F8_{90}$) bias flow groups for each ventilation period. Dots and diamonds represent individual lambs. All data mean and SD. * $p<0.05$, ** $p<0.01$, *** $p<0.001$, **** $p<0.0001$ Tukey post-test (mixed effects model).

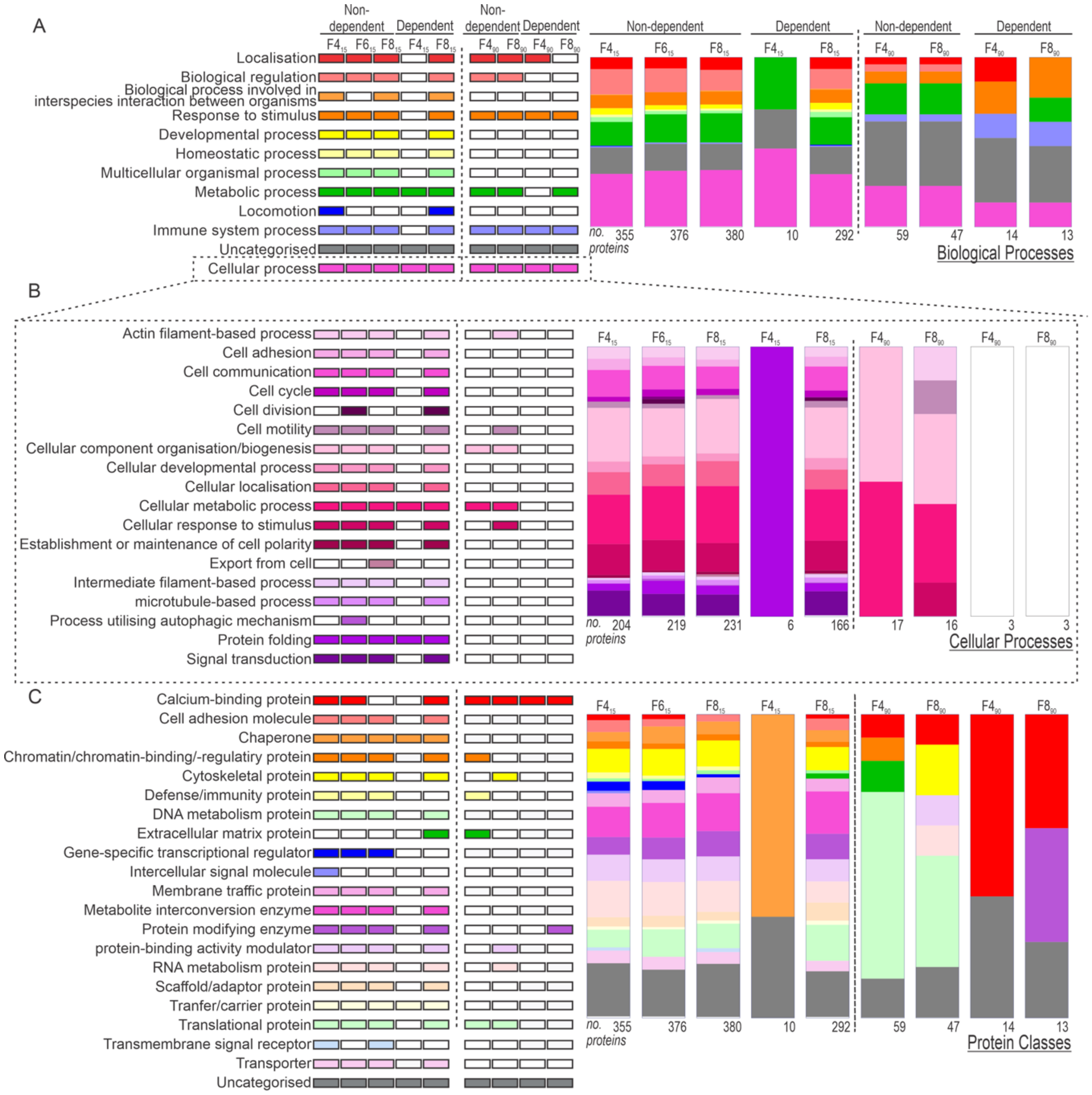

Supplementary Figure E9. PANTHER analysis of differentially expressed proteins identified in non-dependent and dependent lung following 15- and 90-minute flow ventilation strategies. in PANTHER software displaying biological processes (**A**), breakdown of cellular processes (**B**) and protein classes (**C**). Coloured boxes reflect processes that contain >3 DEPs within a ventilation group. Grey boxes represent DEPs that are uncategorised in PANTHER software. Processes with <3 DEPs are not presented.
